# Sequential Effect of Reaction Temperature on Physical and Chemical Properties of ZnO Nanoparticles for Water Purification in a Photocatalytic Reactor

**DOI:** 10.1021/acsomega.5c13202

**Published:** 2026-04-17

**Authors:** Maryam Basit, Sofia Javed, Paul Westerhoff, Iftikhar Hussain Gul, Zeeshan Ali, Muhammad Aftab Akram, Nadia Shahzad

**Affiliations:** 1 Department of Materials Engineering, School of Chemical & Materials Engineering, National University of Sciences & Technology (NUST), H-12, Islamabad 44000, Pakistan; 2 School of Sustainable Engineering and The Built Environment, 7864 Arizona State University, Tempe, Arizona 85287, United States; 3 School of Materials Science and Engineering, Peking University, Beijing 100871, China; 4 Department of Materials Science & Engineering, Pak-Austria Fachhochschule, Institute of Applied Sciences & Technology, Haripur 22650, Pakistan; 5 US-Pakistan Centre for Advanced Studies in Energy (USPCAS-E), National University of Science and Technology (NUST), H-12, Islamabad 44000, Pakistan

## Abstract

This study investigates the influence of reaction temperature on the coprecipitation synthesis of ZnO NPs and correlates their structural and morphological evolution with their photocatalytic performance. ZnO NPs synthesized at five temperatures, namely, 25, 35, 45, 55, and 65 °C, produced wurtzite-phase materials with varying crystallinities in the range of 77–89%. XRD, SEM, EDX, UV–vis spectroscopy, BET surface area measurement, and PL spectroscopy were used to obtain structural and compositional information about the synthesized NPs. The increase in temperature transforms the nanosheet-like structures into quasi-spherical particles with average crystallite sizes of 14–20 nm, as a function of variations in crystal defects and a high concentration of lattice distortions. Optical characterization revealed a red shift in the absorption edges, with a reduction in the bandgap (*E*_g_) to 2.75 eV. PL spectra exhibited a pronounced Stokes shift, confirming defect-mediated emission. The BET analysis revealed a maximum BET surface area of 41.14 m^2^/g at 45 °C, and the pore size distribution range is 1.6–48.82 nm, along with a significant change in zeta (ζ) potential. The photocatalytic activities were then determined in the degradation of Rhodamine B (Rh-B) under visible-light irradiation in a specially designed photocatalytic reactor. ZnO prepared at 45 °C exhibited the best performance, degrading up to 99% of Rh-B within 200 min, corresponding to a reaction rate constant of 0.022 min^–1^. Finally, an optimized ZnO sample was produced at 45 °C and directly introduced into the reactor to immobilize the catalyst, thereby avoiding the release of NPs into water streams and eliminating subsequent recovery steps. In this work, the reaction temperature has been identified as a critical determinant of ZnO defect concentration, morphology, and photocatalytic efficiency, providing a scalable route to high-performance photocatalysts for effective, environmentally friendly water purification systems.

## Graphical abstract

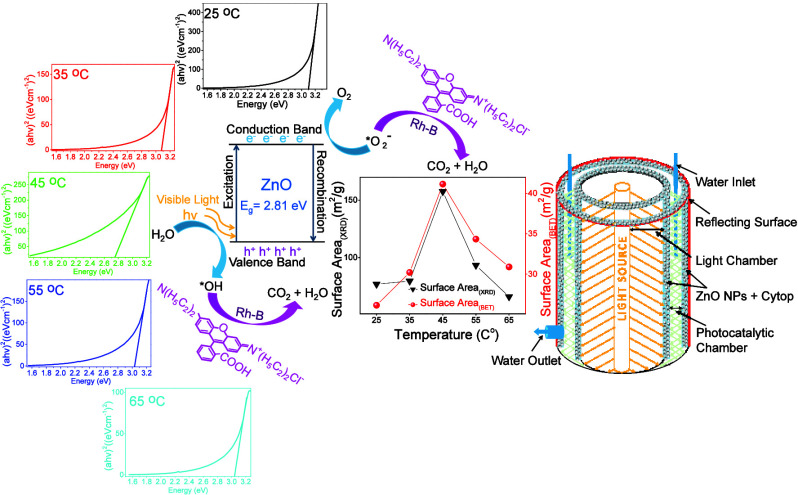

## 1 Introduction

Wide-bandgap (E_g_) metal oxides (MOs) semiconductors have been extensively applied across diverse technological domains. Among them, ZnO has attracted considerable interest owing to its exceptional optoelectronic properties, tunable E_g_, morphological versatility, chemical and thermal stability, intrinsic antibacterial activity, and low production costs, which have enabled it to outperform and replace many other MOs across a wide range of applications. ZnO is an n-type semiconductor with a direct E_g_ of 3.3 eV and a high exciton binding energy of 60 meV, effectively suppressing electron–hole recombination.^1–3^ Its high quantum efficiency further facilitates broad-spectrum light absorption, spanning ultraviolet to visible regions.^4,5^ ZnO nanoparticles (NPs) offer several environmental benefits, such as potential for water purification through photocatalytic degradation of pollutants, sterilization, and antimicrobial activity, reducing reliance on hazardous chemicals.^6^ Therefore, they are vital for maintaining sustainable waste management by increasing the efficiency of pollutant removal in wastewater treatment plants.^7^ Moreover, ZnO NPs are used in biodegradable packaging and antimicrobial coatings,^8,9^ thereby protecting the environment and conserving various resources. In that light, reactive oxygen species (ROS) can be generated when ZnO NPs are illuminated, enabling the degradation of pollutants while offering eco-friendly antimicrobial properties.^8,10^ More importantly, since ZnO is a naturally occurring compound, its toxicity can be minimized through engineering. It can be considered an excellent candidate for greener environmental applications.^11–14^

Due to its environmentally benign nature, rich surface reactivity, and biocompatibility, ZnO has been extensively utilized in pharmaceuticals^15^, medicine^16^, food preservation^17^, optical and gas sensors^18^, transparent conductive thin films,^19,20^ surface coatings,^8^ cosmetics,^21^ and water purification technologies,^22–25^ particularly in photocatalysis.^13,26–29^ Numerous studies have demonstrated ZnO as a highly efficient photocatalyst under UV and visible light irradiation.^30,31^ Its photocatalytic activity is strongly influenced by structural and physicochemical parameters, including particle size, morphology, crystallinity, intrinsic defect density, oxygen vacancies (OVs), and E_g_.^13,26^ Despite these advances in the photocatalytic potential of ZnO, there remains a lack of systematic studies addressing the influence of synthesis temperature on its morphological evolution and photocatalytic performance of ZnO NPs.

Several synthetic approaches have been explored to tailor the desired properties of ZnO, including coprecipitation,^32^ chemical bath deposition,^9^ hydrothermal synthesis,^33^ and green synthesis techniques.^34^ Each method offers varying degrees of control over parameters like E_g_ modulation, crystal defect engineering, morphological variation, and the introduction of OVs.^35,36^ Specific morphologies, crystallite sizes (‘D), and optical and dielectric properties of ZnO nanostructures lend themselves to distinct application.^37^ Notably, ZnO nanostructures enriched with OVs and intrinsic defects demonstrate superior degradation efficiency for organic dyes.^38,39^

Coprecipitation is one of the most widely adopted methods due to its simplicity and effectiveness. It offers precise control over crystallinity, morphology, and surface area while enabling E_g_ tuning by inducing lattice stress.^40,41^ These localized stresses influence the morphology, E_g_, and optical properties of the resulting NPs.^32^ Coprecipitation allows for flexible control of composition, pH, sintering temperature, and morphology at a scalable level. A range of morphologies can be achieved by altering precursors, limiting agents, stabilizers, and annealing conditions. Its cost-effectiveness, scalability, and high purity make coprecipitation a prominent method for industrial-scale synthesis of ZnO.^42,43^

Water treatment for industrial and potable water sources has become a critical global concern due to the persistent organic pollutants (POPs). UN’s Sustainable Development Goals 6 exclusively discussed the necessity of clean water.^44^ Conventional methods, such as biological treatment systems, membrane filtration, carbon adsorption, thermal and chemical oxidation, are often costly and maintenance-intensive and can result in the formation of toxic intermediates.^45,46^ These byproducts necessitate the adoption of advanced oxidation processes (AOPs) for complete degradation. Among AOPs, photocatalysis stands out for its cost-effectiveness, high efficiency, and minimal maintenance. It employs photocatalytic reactions to generate hydroxyl (•OH) radicals capable of decomposing various organic pollutants (OPs) such as dyes.^47–49^ Not only does this reduce the production of intermediate products, but it also ensures more environmentally sustainable methods for POP degradation.

Historically, hematite NPs were employed for this purpose due to their small E_g_ and stable ionic structure, which supported redox reactions.^50,51^ However, their inefficient charge transport led researchers to explore alternatives. ZnO has since become a prominent candidate thanks to its tunable E_g_, improved charge-transport properties, and defect-engineering capabilities.^22,52,53^ The need for clean water remains especially critical in rural and underdeveloped regions, where access to safe filtration systems is limited. OPs leaching into underground water supplies and entering primary water streams pose significant health risks.

In 2022, elevated levels of POPs were reported in groundwater across multiple regions,^54–56^ leading to the classification of these water sources as unfit for human use. The increasing concentration of POPs has raised concern among pediatricians and public health experts about its impact on local agriculture.^57^ Beyond environmental contamination, these pollutants threaten human health, including neurological, intelligence quotient, and genetic damage. Recent studies have shown a concerning correlation between long-term exposure of human life to these organic contaminants and reduced antioxidant levels in children, along with genetic mutations that lead to oxidative stress and muscular degeneration.^55,56^ These alarming findings emphasize the need for portable, accessible water purification systems. In this study, we developed a prototype of a portable photocatalytic reactor capable of degrading OPs under visible light.

Numerous studies have focused on the effects of annealing and calcination on ZnO NPs^58,59,24^ and investigated the photocatalytic properties.^40^ M.G. Kotresh et al. conducted a study to examine the effects of reaction temperatures at 50 and 70 °C using the coprecipitation method with Zn­(NO_3_)_2_·6H_2_O as the precursor.^60^ Another study was conducted by S.Y. Purwaningsih et al. to investigate the effects of reaction temperature (85 °C) and pH, using a 0.5 mol/L HCl solution to dissolve Zn­(CH_3_COO)_2_·2H_2_O.^61^ U. Manzoor et al. conducted a study on ZnO synthesized by the coprecipitation method in an alcoholic medium at 60 °C, 63 °C, and 65 °C.^62^ The solubility of zinc-based salts is strongly affected by pH. Moreover, each basic group in a metallic salt has a unique effect on the salt’s solubility. However, the role of reaction temperature in coprecipitation synthesis has not been systematically studied, as it is often overlooked alongside changes in precursors, solution pH, heating duration, and nucleation time. In this article, we investigated the effects of controlled reaction temperature over a wide range during the synthesis of ZnO NPs using the coprecipitation method while maintaining uniformity in all other synthesis parameters. Indeed, this focused approach enabled the isolation of only those temperature driven intrinsic modifications in ZnO, such as defect-mediated E_g_ narrowing, altered photonic absorption, and lattice strain (ε), without confounding external pH adjustments.

Although it is well established in photocatalysis that pH can have a profound impact on dye degradation kinetics, as it may perturb the protonation–deprotonation equilibrium and alter dye adsorption, surface charge, and ROS generation, considering the vast amount of literature that describes pH-dependent behavior of cationic dyes on ZnO surfaces.^25,40^ Rhodamine B (Rh–B) was selected as a model organic pollutant due to its complex molecular structure, chemical stability, and strong chromophore, making it an ideal probe for evaluating photocatalytic degradation efficiency under visible-light irradiation. Its distinct absorption peak (∼554 nm) enables precise spectrophotometric monitoring of degradation kinetics and the formation of intermediates, providing a reliable benchmark for photocatalyst performance.^33,63^ Furthermore, Rh–B is a widely used xanthene dye in textiles, cosmetics, and biological staining; its persistence and potential carcinogenicity make it a representative contaminant in real industrial wastewater.^64^ Thus, its degradation not only serves as a standard for mechanistic evaluation but also demonstrates the practical applicability of ZnO-based photocatalysts for environmental remediation. In this regard, the utilization of Rh–B as a model dye pollutant is consistent with recent studies assessing the photocatalytic performance of ZnO nanostructures.^33,63,65–67^

The prepared NPs were then used to degrade Rh–B, a model dye, in tap water without the use of any additional sacrificial agent. Finally, the best-performing ZnO NPs were used to design a photocatalytic reactor. The effects of reaction temperature have led to significant variations in the properties of the ZnO NPs. The pronounced morphological shift, ‘D, intrinsic defects, lattice distortions consistent with defect formation, and E_g_ are significant factors affecting photocatalytic activity. This study will contribute to a better understanding of interfacial activity and the physical and chemical properties of ZnO NPs. Moreover, it will help researchers reproduce large scale ZnO nanostructures using the coprecipitation method. Furthermore, the study has been extended by designing a novel prototype of a visible light active photocatalytic reactor using the best-performing ZnO NPs from the photocatalytic experiments. The reactor confines the flow of ZnO NPs in the mainstream water systems, eliminating the need for a complex post recovery process of NPs from the system.

## 2 Experimental Section

### 2.1 Chemicals and Materials

Zinc acetate dihydrate (Zn (CH_3_CO_2_)_2_·2H_2_O, 99% purity) and triethylamine (TEA, N­(CH_2_CH_3_)_3_, 99% purity) were procured from Riedel-de Haen, while sodium hydroxide (NaOH, 98% purity) was sourced from Daejung Chemicals. All reagents were used as received without further purification. Rhodamine B (Rh–B, C_28_H_31_ClN_2_O_3_), the model cationic dye for photocatalytic degradation studies, was obtained from Alfa Aesar with a stated purity of 98%.

The prototype photocatalytic reactor was assembled using a polished quartz glass tube as the inner cylinder with a diameter of 7.5 cm, manufactured by Liling Xing Tai Long Special Ceramic Co., Ltd. (Model: XTL), and an acrylic outer cylinder with a diameter of 11.5 cm supplied by Guangzhou Hengge Plastic Products Co., Ltd. (Model: AT-1). A hydrophobic fluoropolymer coating of Cytop (Product No. CTX 109AE) was purchased from AGC Inc. to minimize surface fouling. Illumination was provided by a Philips TL 20*W*/52 SLV/25 fluorescent lamp (Product Code: 871150064302540), integrated within the reactor.

### 2.2 Synthesis of ZnO Nanoparticles (NPs)

A 0.2 M aqueous solution of Zn­(CH_3_CO_2_)_2_·2H_2_O was prepared using double-deionized water. Subsequently, 100 μL of TEA was added, followed by dropwise addition of a freshly prepared 5 M NaOH solution until the mixture reached pH 10, as measured with a calibrated digital pH meter.

Zn^2+^ and OH^–^ ions are produced in an aqueous solution of Zn (CH_3_CO_2_)_2_·2H_2_O through the controlled addition of NaOH. This initiates Zn­(OH)_2_ nuclei, which undergo aging and controlled thermal treatment to yield ZnO NPs.^32^ The formation of white precipitates confirmed the nucleation of ZnO NPs. The reaction mixture was stirred continuously and vigorously for 30 min while maintaining a constant reaction temperature in a thermostatically controlled water bath at 25 °C, 35 °C, 45 °C, 55 °C, and 65 °C.

Following synthesis, the mixtures were held at 60 °C for 19 h to stabilize. The precipitated ZnO NPs were then thoroughly washed with deionized water and subsequently annealed at 400 °C for 2 h in a muffle furnace. Previous studies have established that annealing temperature, stabilization duration, and synthesis pH significantly influence the morphology, crystallinity, and photocatalytic performance of ZnO NPs.^68^ These parameters were preoptimized and carefully maintained throughout the experiment to ensure that the only variable in this study is the reaction temperature, thereby ensuring reproducibility and consistency across all samples. Coprecipitation ensures homogeneous nucleation, controlled growth, and uniform particle distribution, unlike mere hydrolysis, sol–gel, and other synthesis methods. The coprecipitation method has been found to produce NPs with control over size, crystallinity, and defect density, a key optimization for enhancing photocatalytic performance.^32,59,69^

### 2.3 Photocatalytic Activity

Rh–B was employed as a probe organic pollutant to study the photocatalytic performance of the ZnO NPs. All photocatalytic degradation experiments were conducted under visible light at ambient temperature and pressure. Twenty mg of ZnO photocatalyst was added to 50 mL of an aqueous Rh–B solution (5 mg/L), prepared with double distilled water. The catalyst loading of 20 mg in 50 mL (0.4 g L^–1^) was selected to ensure sufficient surface active sites and stable pseudo first order kinetics, consistent with the optimized range reported for Rh–B–ZnO systems is of ∼ 0.2–0.5 g L^–1^ to achieve high photodegradation efficiency while maintaining linear kinetic regimes in ZnO-based photocatalytic reaction.^70,71^ Photocatalytic parameters, including pollutant concentration, catalyst loading, irradiation time, pH, temperature, and light intensity calibration (e.g., 100 W m^–2^ ≈ 0.1 sun), were optimized based on the reported literature ranges for Rh–B degradation using ZnO photocatalysts.^64,70,72^ The selected conditions minimized light scattering and aggregation effects, ensuring that the observed variations in degradation efficiency accurately reflected the intrinsic influence of synthesis temperature on ZnO NPs properties under visible light irradiation.^71^

The suspension was vigorously stirred magnetically and maintained in the dark for 30 min to establish equilibrium. Following the equilibration period, the mixture was exposed to visible light, with the light source positioned 3.5 cm above the reaction mixture. The degradation of Rh–B was monitored at 25 min intervals over a total irradiation period of 200 min. Samples were withdrawn at each interval and analyzed spectrophotometrically to determine the residual dye concentration. The photocatalytic percentage degradation efficiency (%) was calculated using eq 1:^73^

$$\%\mathrm{age}\;\mathrm{Decay}\;\mathrm{Efficiency}={\frac{\left(C_{o}-C_{t}\right)}{C_{o}}}^{*}100$$ 1

Where C_o_ is the equilibrium concentration of Rh–B (mg/L) prior to light exposure, and C_t_ is the Rh–B concentration (mg/L) at a given irradiation time t. The visible-light source for both photocatalytic studies and reactor performance evaluation was a visible range fluorescent lamp, emitting in the 400–500 nm wavelength range. The product matches the optical absorption characteristics of the synthesized ZnO NPs obtained at different synthesis temperatures, thus providing equal excitation across the samples. The incident light intensity was maintained at 100 W m^–2^ (≈ 0.1 sun) to ensure consistent photon flux and minimize variability across experimental runs of photocatalytic degradation, reactor functionality, and stability tests.

### 2.4 Photocatalytic Reactor Design

A prototype visible-light-active photocatalytic reactor was developed using ZnO NPs synthesized at 45 °C, chosen for their superior photocatalytic performance. The reactor comprises a dual-cylinder configuration: the inner chamber is constructed from polished quartz glass (7.5 cm in diameter) to minimize light absorption in the visible range. The outer cylinder (11.5 cm diameter) is fabricated from acrylic and internally lined with a 2 mm flexible mirror sheet to maximize light reflection and internal scattering.

Both the outer surface of the quartz tube and the inner surface of the mirrored acrylic chamber were spray-coated with a composite layer comprising ZnO NPs and Cytop, a perfluorinated polymer (perfluoro (1-butenyl vinyl ether)) at a 9 wt % loading. The uniformity of this hybrid coating was maintained at approximately 5 μm to balance photocatalytic surface activity with optical transparency. The chamber width was optimized to 1.4 cm to mitigate light attenuation, with the light source placed at a fixed distance of 3.5 cm from the reactor. The reactor’s length was 60 cm, with an outlet 5 cm from the base. The selected light source emits within the visible range, aligning with the ZnO NPs optical E_g_ (2.75 eV), corresponds to a response wavelength of ∼ 443 nm, facilitating visible-light-induced photocatalysis. Contaminated water containing Rh–B dye (5 mg/L concentration) was introduced into the photocatalytic chamber, where it was continuously exposed to visible irradiation for 3 h per cycle. Figure 1 shows a schematic diagram of a visible-light-active photocatalytic reactor prototype.

**Figure 1:**
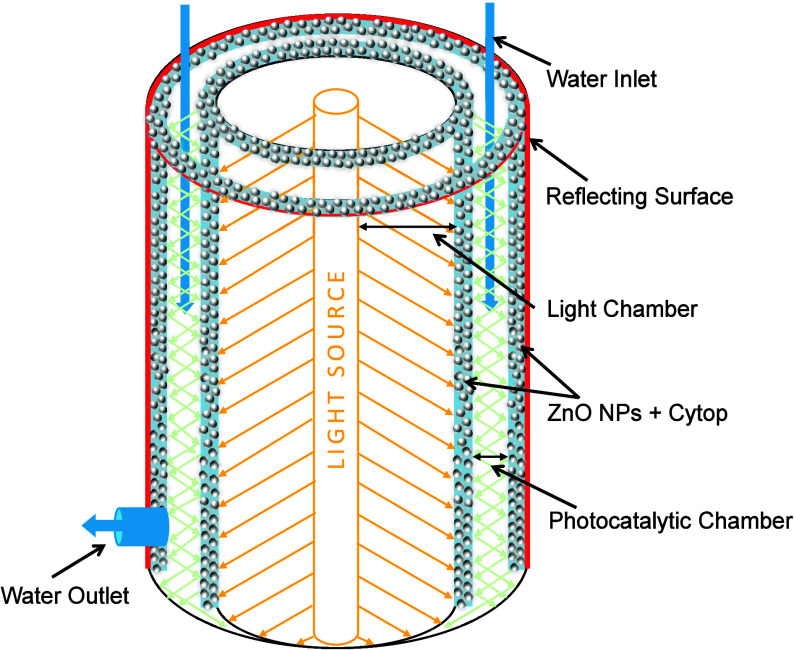
Schematic of the visible-light photocatalytic reactor integrating with ZnO NPs. The dual-cylinder design enhances photon utilization through internal reflection and stable coating of NPs.

### 2.5 Characterization of the Fabricated ZnO Samples

A comprehensive suite of analytical techniques was employed to investigate the structural, morphological, optical, and surface properties of the synthesized ZnO NPs. The surface morphology and elemental composition were examined using scanning electron microscopy (SEM) and energy-dispersive X-ray spectroscopy (EDX) on a JEOL JSM-6490-A system. Crystallographic analysis was conducted via X-ray Diffraction (XRD) using a STOE Siemens D5005 diffractometer with Cu Kα radiation (λ = 1.5406 Å) to determine the crystal phase and lattice structure.

Specific surface area measurements were carried out using Brunauer–Emmett–Teller (BET) analysis on a Micromeritics Gemini VII 2390 surface area analyzer. To gain insight into the electronic and defect states of the ZnO nanostructures, photoluminescence (PL) spectroscopy was performed using a FluoroMax-4 spectrofluorometer (Horiba Scientific, Japan). Optical absorption characteristics were recorded using a Jenway 72-series UV–visible spectrophotometer. This instrument was also employed to monitor the photocatalytic degradation of Rh–B dye in the UV–vis spectral range.

## 3 Results and Discussion

### 3.1 Scanning Electron Microscopy (SEM)

Figure 2 presents SEM micrographs of ZnO NPs synthesized at 25 °C, 35 °C, 45 °C, 55 °C, and 65 °C. A notable morphological transition is observed with temperature increases. At 25 and 35 °C, the ZnO structures exhibit nanosheet like morphologies characterized by multiple sheets radiating from a single nucleation center. As the synthesis temperature reaches 45 °C, a distinct shift in morphology occurs, with the nanostructures transitioning to quasi spherical forms. The reported SEM micrographs were analyzed using “ImageJ”; statistical averages of lateral dimensions of nanostructures are represented in Table 1 rather than actual crystallographic thickness.

**Figure 2:**
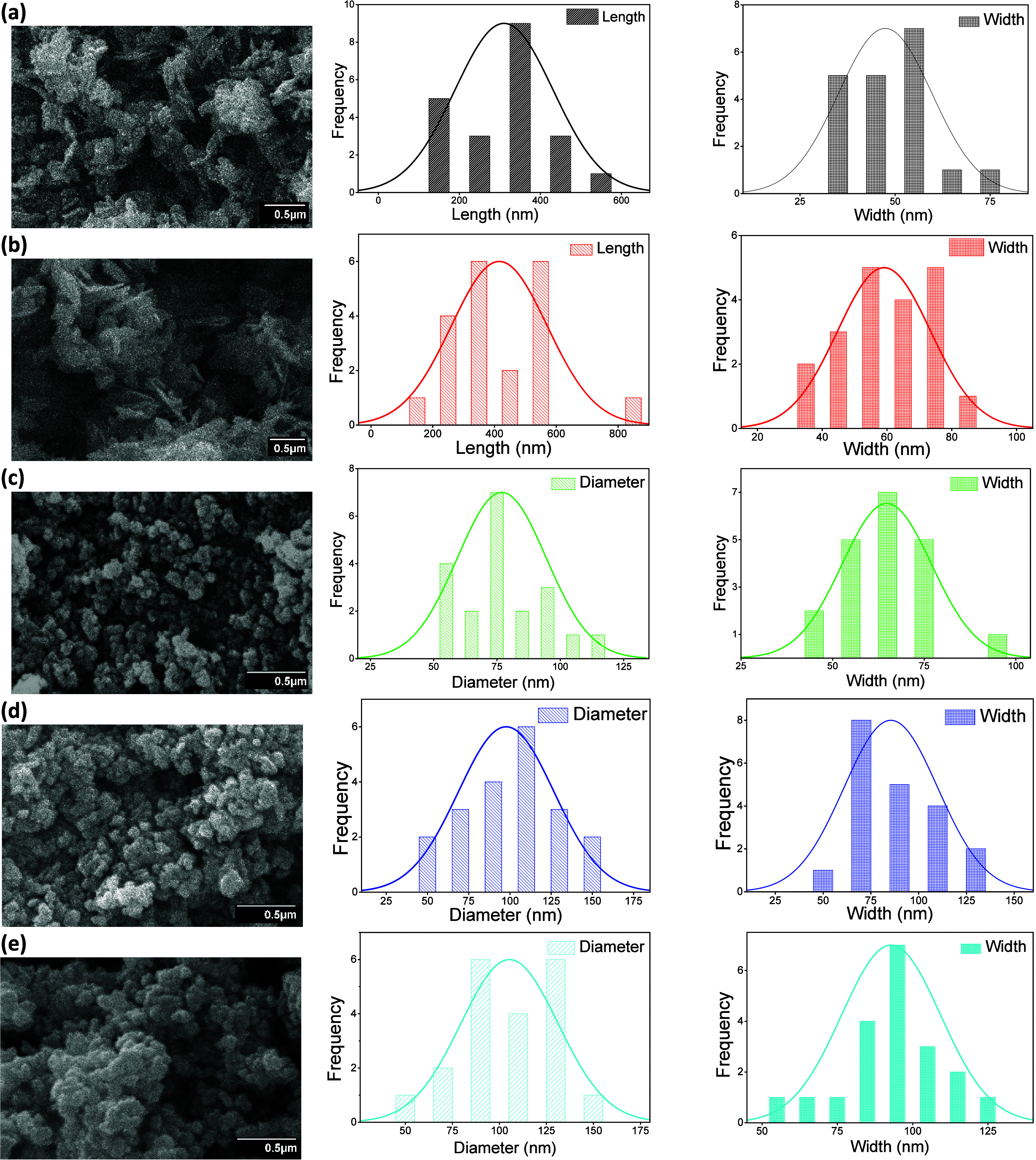
SEM micrographs with the histogram of both length and diameter of ZnO NPs exhibiting the morphology.

**Table 1:** Effect of Synthesis Temperature on the Average Morphological Dimensions of ZnO NPs

**temp during synthesis**	**length (nm)**	**width (nm)**	**morphology**
25 °C	310.28 ± 5	47.42 ± 5	nanosheets
35 °C	415.45 ± 5	59.1 ± 5	nanosheets
45 °C	76.9 ± 5	64.8 ± 5	quasi-spherical NPs
55 °C	97.75 ± 5	85.45 ± 5	quasi-spherical NPs
65 °C	105.35 ± 5	92.85 ± 5	quasi-spherical NPs

The smallest grain size was observed at 45 °C, indicating a higher grain boundary-to-surface area ratio. The small particle size at 45 °C is presumed to confer a higher surface-active site density, which could lead to better photocatalytic activity. With a further increase in temperature (from 55 to 65 °C), particle sizes begin to increase, suggesting the onset of crystal coalescence and the formation of a polycrystalline structure. Despite identical postsynthesis thermal treatment, there are notable morphology variations between ZnO NPs synthesized at different reaction temperatures, implying that initial nucleation conditions have a lasting influence on determining the final nanostructure. The temperature dependent morphological transformation (Table 1) demonstrates that thermal energy input plays a crucial role in the nucleation and growth kinetics of NPs. The results are well within the reported literature on thermally controlled ZnO nanostructure preparation.^37,64^

In the coprecipitation synthesis, temperature directly controls supersaturation level, ion mobility, precursor hydrolysis, and reaction kinetics, which, in turn, control the nucleus formation rate and facet-specific growth before annealing.^37,64,72^ Although precursor concentration has varied across studies on this material system, it is kept constant across all synthesized samples; hence, temperature remains the dominant variable influencing ion mobility and, thereby, the effective supersaturation in the present system.

Lower synthesis temperatures, such as 25 and 35 °C, result in sheet-like morphologies due to slow reaction kinetics and poor precursor diffusion. Anisotropic growth was preferred under these conditions and was driven by selective adsorption on specific crystal facets, which impeded growth along the *c*-axis while promoting lateral growth. According to the literature, lower temperatures support anisotropic growth through selective adsorption onto polar surfaces, whereas at higher temperatures, it changes to isotropic crystallization combined with aggregation into compact morphologies.^37,74^

In contrast, at elevated reaction temperatures (45 °C and above), the thermal energy enhances atomic mobility and nucleation rates. resulting in a shift in the growth mechanism toward isotropic crystallization and leading to the formation of quasi-spherical particles that are thermodynamically more stable due to their minimized surface-to-volume ratio and lower surface energy. Furthermore, higher temperatures also promote higher supersaturations in the reaction mixture. This results in more nucleation sites. Also, higher temperatures lead to more uniform NP growth due to higher supersaturation. The frameworks formed in the initial reaction phase through nucleation are maintained through thermal conversion.^75^

The observed SEM images of the morphological transition from nanosheets to quasi spherical nanostructures are attributed to the interplay between kinetic control of the synthesis reaction at low temperatures and thermodynamic dominance at higher temperatures. While SEM does not directly resolve crystallographic facets, growth directions, and surface chemical states, the observed morphological evolution is consistent with temperature dependent thermodynamic growth previously reported for ZnO coprecipitation.^74,75^ SEM effectively visualizes overall surface topographical morphology and agglomeration states.

### 3.2 X-ray Diffraction (XRD)

The XRD patterns of the synthesized ZnO NPs by the coprecipitation method at different synthesis reaction temperatures are shown in Figure 3a. The clear, sharp peaks observed in the diffractograms indicate the existence of highly crystalline structures, scanned by Cu Kα radiation (λ = 1.5406 Å). The observed peaks are identified as the hexagonal wurtzite structure of ZnO (JCPDS No. 96–900–4180).^76–78^ The planes (100), (002), (101), (102), (110), (103), (112), and (201) are located at 2θ values of 31.74°, 34.42°, 36.22°, 47.52°, 56.54°, 62.84°, 67.89°, and 69.02°, respectively. The peak strength increases up to 35 °C, indicating high crystallinity. Increasing the synthesis temperature to 45 °C and above reveals a slight increase in the 2θ values of the observed peaks in the crystal structures shown in Figure 3a. The systematic nature of the peak shifts across multiple reflections suggests ε-induced lattice distortion rather than instrumental artifacts, consistent with temperature-driven growth kinetics. Coinciding with the morphological transformation observed in the SEM images, indicating the development of tensile ε and crystal defects within the ZnO crystal lattice under various thermal growth conditions. These shifts have been previously attributed to changes in ‘D, crystallite defects, lattice distortions, internal stress, and tensile ε.^79,80^ It is caused by increased atomic oscillations and the initiation of nucleation at multiple sites at higher temperatures, where increased thermal energy accelerates nucleation and growth. This stress, along with lattice distortion, affects the peak position and intensity, indicating changes in the crystal structure.^24,38^

**Figure 3:**
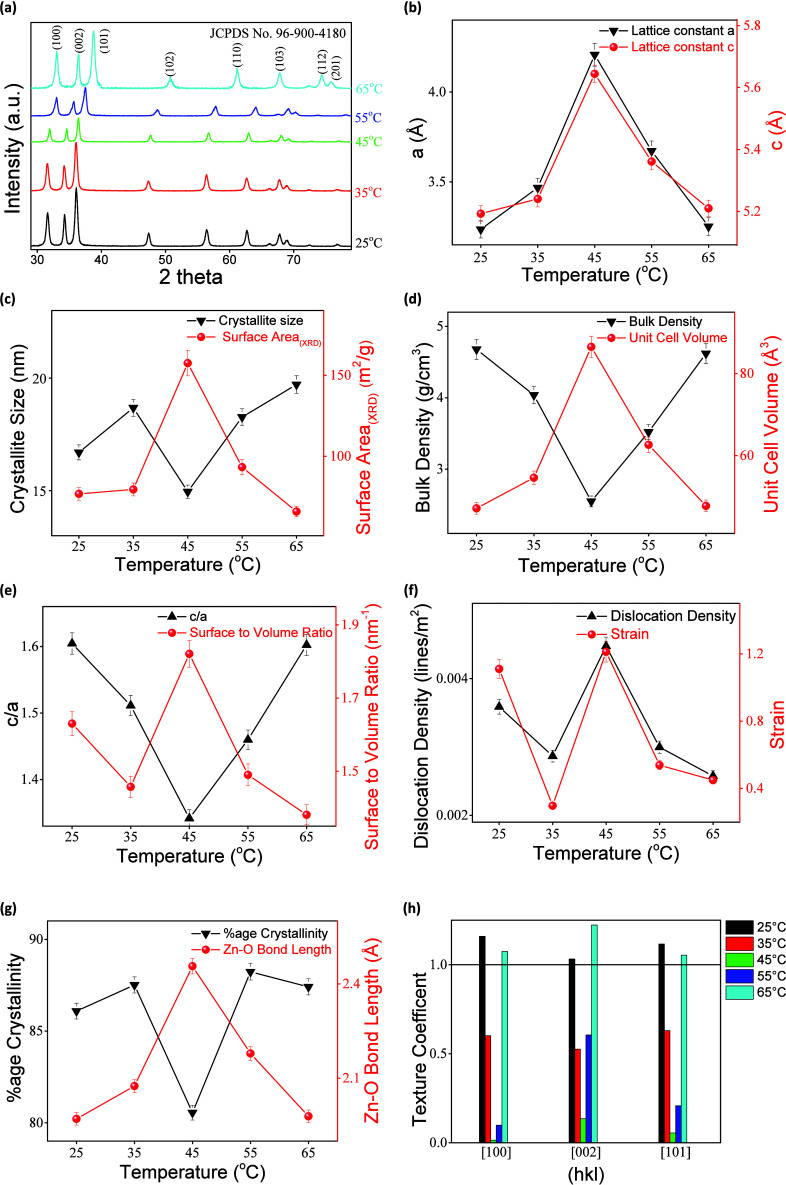
(a) XRD patterns of ZnO NPs prepared at different synthesis temperatures via the coprecipitation method. Variation of structural parameters of ZnO NPs with synthesized at different reaction temperatures: (b) lattice constants a and c; (c) ‘D and XRD surface area (S_(XRD)_); (d) bulk density (ρ) and unit cell volume (V); (e) c/a ratio and surface-to-volume ratio; (f) dislocation density (ρ_(dislocation)_) and lattice strain (ε); (g) crystallinity and Zn–O bond length (L_(Zn–O)_), (h) Texture coefficient (TC _(hkl)_) of ZnO NPs along (100), (002), and (101) planes.

In addition to this, the lower peak intensity at 45 °C also denotes that there is an elongation of the crystal lattice along the *c*-axis because of a drastic change in lattice constant, which denotes the formation of a wurtzite ZnO structure. The elongation along the *c*-axis indicates changes in binding and coordination, which affect the anisotropic crystal growth and morphological evolution. This is a structural property that is important in creating different shapes of ZnO NPs, such as nanosheet and quasi-spherical NPs.^18^

Figure 3b illustrates the variation in the lattice parameters *a* and *c* of ZnO NPs as a function of growth temperature. The lattice parameters were calculated in detail to further elucidate the impact of temperature on the crystallographic structure. The lattice constants “a” and “c” were extracted using the corresponding reflection planes by XRD data analysis. ZnO has a hexagonal wurtzite structure (space group P63mc), characterized by lattice parameters in which a = b ≠ c, and angles α = β = 90° and γ = 120°. Equations 2 through 5 were used to compute a_(100)_ and c_(002)_. Allowing for a detailed assessment of structural evolution as a function of synthesis temperature.^81^

$$\frac{1}{d_{\left(hkl\right)}^{2}}=\frac{4}{3}\left(\frac{h^{2}+hk+k^{2}}{a^{2}}\right)+\frac{l^{2}}{c^{2}}$$ 2

$$a_{\left(100\right)}=\sqrt{\frac{4d_{\left(100\right)}^{2}}{3}};c_{\left(002\right)}=2d_{\left(002\right)}$$ 3

$$n\lambda =2d_{\left(hkl\right)}\mathrm{Sin}\theta_{\left(hkl\right)}$$ 4

$$a_{\left(100\right)}=\frac{\lambda }{\sin\theta_{\left(100\right)}\sqrt{3}};c_{\left(002\right)}=\frac{\lambda }{\sin\theta_{\left(002\right)}}$$ 5

Where *h*, *k*, and *l* are the Miller indices, *n* denotes the order of diffraction (taken as 1 for the first-order maxima), λ is the wavelength of the incident X-rays (Cu Kα = 1.5406 Å), and θ_(100)_ and θ_(002)_ represent the diffraction angles corresponding to the (100) and (002) planes, respectively.^81^ There is a marked increase in the magnitudes of both lattice constants at 45 °C, but a decrease at higher synthesis temperatures. The sudden change in the XRD pattern at 45 °C directly corresponds to the observed transition in particle morphology in the SEM images, indicating an apparent covariance between the crystal structure and particle morphology. The above observations confirm the previous finding that the reaction temperature has a profound effect on the crystal structure and morphology of the synthesized ZnO NPs, due to lattice ε and anisotropic crystal growth.

Figure 3c illustrates the inverse relationship between the ZnO NPs surface area (S_(XRD)_) and crystallite size (‘D). As is well documented, a reduction in ‘D is accompanied by a significant increase in specific surface area. Scherrer’s Equation (eq 6) computes the ‘D.

CrystallineSize(D‘)=kλβcos⁡θ 6

The surface area (S_(XRD)_) of synthesized ZnO NPs was calculated by eq 7.

SurfaceArea(S(XRD))=6×103D‘×ρ 7

In eq 7, effective S_(XRD)_ is inversely proportional to the ZnO ‘D and bulk density (ρ*)*. An enhanced S_(XRD)_ indicates more reactive sites on the particle surface, and increased defects facilitate photocatalytic redox reactions more efficiently. Variations in measured lattice constants from the values of bulk ZnO indicate the presence of temperature-induced lattice distortion, reflected in internal lattice stress, ε, and ‘D values. Such variations affected the Zn–O bond length (L_(Zn–O)_), which has been listed in Table 2.

**Table 2:** Summary of Crystallographic Parameters of ZnO NPs Synthesized at Varying Reaction Temperatures, including Lattice Constants (a, c), Crystallite Size (‘D) (Scherrer), Microstrain (ε), Bulk Density (ρ), and Zn–O Bond Length (L_(Zn–O)_)

	**lattice constant**		**crystallite size (**‘D**)**	**surface area** (S_(XRD)_)	**bulk density** (ρ*)*	**unit cell volume (**V**)**		**Zn–O bond length** (*L*_*(Zn*–*O)*_)
**temp during synthesis**	**a (Å)**	**c (Å)**	**(nm)**	**(m^2^/g)**	**(g/cm^3^)**	**(Å** ^**3**^ **)**	**microstrain (ε)**	**(Å)**
25 °C	3.24	5.19	16.69 ± 5	76.79	4.68	47.11	1.11	1.98
35 °C	3.47	5.24	18.68 ± 5	79.53	4.04	54.58	0.29	2.08
45 °C	4.21	5.64	14.95 ± 5	157.52	2.55	86.52	1.21	2.46
55 °C	3.67	5.36	18.27 ± 5	93.3	3.52	62.62	0.54	2.18
65 °C	3.25	5.21	19.71 ± 5	65.89	4.62	47.71	0.45	1.98

The ρ of ZnO NPs was calculated using eq 8:

$$\mathrm{Bulk}\;\mathrm{Density}\left(\rho \right)=\frac{\mathrm{nM}}{N_{A}V}$$ 8

Here, n is the number of atoms in each unit cell (n = 6 for a hexagonal cell in the wurtzite structure), M is the molar mass of ZnO (81.38 g/mol), N_A_ is Avogadro’s number (6.023 × 10^23^ mol^–1^), and V is the unit cell volume (*V*).^82,83^ The values of ρ obtained for the prepared ZnO NPs were considerably lower than that of bulk ZnO; this is because lower density is attributed mainly to the increment in pore volume and the decrement in pore number,^84,85^ implying that the synthesis temperature exerts a direct effect on the pore structure, accordingly influencing the values of ρ and S_(XRD)_.

Figure 3d shows that ZnO NPs synthesized at 45 °C exhibit the lowest ρ, corresponding to the largest calculated *V* associated with effective photocatalytic degradation. The *V* was determined using eq 9.^81^

$$\mathrm{Unit}\;\mathrm{Cell}\;\mathrm{Volume}\left(V\right)=\left(\frac{\sqrt{3}}{2}\right)a^{2}c$$ 9

As the reaction temperature increases, the average kinetic energy increases, reducing the time required for Zn and O atoms to attach to specific nucleation sites for crystal growth. This will cause lattice disorder, leading to imperfections in the ZnO crystal lattice. Therefore, there will be an increase in V at 45 °C, despite the decrease in ‘D, which continues to decrease with increasing reaction temperature. The changes in the crystal structure are consistent with those observed in morphology at 45 °C, where the ZnO NPs changed from nanosheet to quasi-spherical structures. The V expansion can be attributed to lattice ε, surface stress associated with nano crystallites, and kinetic disorder during rapid nucleation. With increasing temperature, the nucleation points shift, leading to isotropic growth and, consequently, lower V due to lower defect concentrations.^37,72^

Figure 3e displays a comparison of the c/a ratio and surface-to-volume ratio for ZnO NPs prepared at different temperatures. The increase in the surface-to-volume ratio is directly proportional to enhanced photocatalytic activity, owing to the greater number of surface-active sites that facilitate redox reactions. It is also observed that the c/a ratio for the ZnO NPs prepared at 45 °C is the lowest, indicating a reduced difference in the lattice constants c and a and thus minimizing anisotropic growth.

The occurrence of abrupt lattice distortion and morphological change at 45 °C indicates a remarkable coupling between the lattice structure and crystal growth during the temperature modulated synthesis. This lattice symmetry deviation leads to a remarkable increase in the density of structural defects, which act as key factors in preventing recombination during the photocatalytic reaction. On the other hand, the shifting and broadening of the diffraction peaks of the XRD patterns, especially those of the smaller-NPs (<100 nm), are indicative of lattice ε, which often occurs due to size-induced stress.^37^ A general trend is observed where an increase in ‘D corresponds to a decrease in internal ε. The ε in the ZnO crystal lattice was quantitatively measured using eq 10.^81^

$$Strain\left(\varepsilon \right)=\frac{\beta_{\left(hkl\right)}}{4\tan\theta_{\left(hkl\right)}}$$ 10

Dislocations are irregularities in the crystal structure and are also important defects in photocatalytic reactions. The dislocation density **(ρ**_**(dislocation)**_ **)** in the lattice structure is measured by eq 11.^86^

$$Dislocationdensity\left(\rho_{\left(dislocation\right)}\right)=\frac{1}{D^{2}}$$ 11

Figure 3f shows that the synthesized ZnO NPs at 45 °C have the largest ρ_(dislocation)_ and lattice ε values among the prepared samples. This higher defect density translates into larger structural perturbations, in agreement with the sudden morphological change occurring at this temperature. At higher synthesis temperatures, ρ_(dislocation)_ and tensile ε decrease, indicating that the increases in crystallinity and structural expansion occur due to increased atomic mobility and improved NPs growth. The calculated value of ρ_(dislocation)_ provides a relative estimate of lattice imperfections associated with defects, rather than an absolute value, and exhibits a maximum at 45 °C, which correlates with the calculated morphological transition. Furthermore, the lattice ε value of ε, as well as ρ_(dislocation)_, exhibits a deformation of the ZnO lattice structure. Although XRD cannot provide an estimation of OV concentration, deformations of the lattice structure can be ascribed to lattice imperfections or OV defects for kinetically limited synthesized ZnO.

Figure 3g provides information on the crystallite quality in this material, based on comparisons of crystallinity **(%)** and L_(Zn–O)_. The two values provide information about the level of order in this material and its inherent defects. The L_(Zn–O)_ is given by eq 13; in this equation, u′ is the positional parameter in the wurtzite structure of ZnO. The u′ is used to show the difference in positioning between Zn and O in the ZnO NPs structure along the *c*-axis, as it is quite sensitive to deformation in ZnO NPs under processing conditions.^86,87^ Bond length and crystallinity percentage variations directly correlate with defect density and the degree of lattice deformation induced by synthesis conditions.

$$Positional\;parameter\left(u^{\prime }\right)=\frac{a^{2}}{3c^{2}}+0.25$$ 12

$$Bond\;Length\left(L\right)=\sqrt{\frac{a^{2}}{3}+c^{2}{\left(\frac{1}{2}-u\right)}^{2}}$$ 13

The elongation of L_(Zn–O)_ is due to the existence of tensile ε in the crystal lattice structure of the synthesized ZnO NPs. Lattice distortion is more pronounced at higher reaction temperatures; hence, there is a continuous increase in crystallinity, except in the 45 °C sample, where the sudden morphological and structural transitions result in a decrease in crystallinity. The lattice ε, ‘D, and u′ are identified as the most important recovered structural parameters in determining morphology; in turn, variations in bond length and density are considered secondary due to lattice distortion. The percentage crystallinity is obtained from eq 14.

$$\%\mathrm{age}\;\mathrm{Crystallinity}=\frac{Area\;\mathrm{of}\;crystalline\;peaks}{Area\;\mathrm{of}\;all\;peaks}\times \left(100\right)$$ 14

Figure 3h presents the texture coefficient (TC_(hkl)_), which provides insight into the preferred crystallographic orientation of the ZnO crystallites. A value of TC_(hkl)_ = 1 indicates a random orientation of grains, whereas TC_(hkl)_ < 1 suggests a deficiency of orientation along the specified (hkl) plane. Conversely, TC_(hkl)_ > 1 denotes a pronounced preferential orientation in the corresponding crystallographic direction ⟨hkl⟩. An increase in TC_(hkl)_ thus implies enhanced periodicity and alignment within the crystal lattice, which may influence properties such as electron transport, surface reactivity, and anisotropic growth behavior.^51^

The TC _(hkl))_ eq 15 does measure,

Texturecoefficient(TC(hkl))=I(hkl)Io(hkl)N−1∑nI(hkl)Io(hkl) 15

Where I_(hkl)_ represents the relative intensity of a given diffraction plane from the XRD pattern of the sample, I_o(hkl)_ denotes the corresponding intensity from the standard JCPDS reference, *N* is the number of diffraction peaks analyzed, and *n* is the number of reflections considered in the calculation of the TC_(hkl)_.^81,86^ TC_(hkl)_ at 45 °C is reduced, reflecting disrupted preferential growth and leading to enhanced exposure of mixed crystallographic facets, which may increase the density of surface-active sites relevant to photocatalysis.

Table 3 compiles the TC_(hkl)_ for the (100), (002), and (101) planes, emphasizing the most favored crystallographic directions for ZnO NPs synthesized at different growth temperatures. Figure 3h shows that, in all cases, (002) retains a high TC_(hkl)_ value, establishing a strong crystallographic texture along the *c*-axis in the wurtzite hexagonal structure of ZnO NPs. A significant decrease in TC_(hkl)_ values for all three planes is observed for a specimen grown at 45 °C, indicating a lack of crystallinity and favored crystal growth. A decrease in the TC_(hkl)_ value indicates a lack of nucleation and crystal alignment with a more disordered crystal structure plagued by imperfections such as lattice distortion and dislocations. Conversely, although charge-carrier dynamics are not explored in XRD analysis, large values of lattice ε and reduction in ‘D have been shown to affect defect-related charge-trapping mechanisms. Such a phenomenon can be inferred by a decrease in PL brightness noticed for the specimen at 45 °C.

**Table 3:** Texture Coefficient (*TC*_*(hkl)*_) Values of ZnO NPs along the (100), (002), and (101) Crystallographic Orientations at Varying Synthesis Temperatures, Highlighting the Temperature-Dependent Evolution of Preferred Crystal Growth Directions

**(hkl)**	**25 °C**	**35 °C**	**45 °C**	**55 °C**	**65 °C**
**(100)**	1.15921	0.60113	0.01399	0.09817	1.07431
**(002)**	1.03165	0.52527	0.13476	0.60526	1.22277
**(101)**	1.11649	0.62954	0.05439	0.20733	1.05339

It is important to emphasize that XRD characterization is a volumetric of crystallography, and the data obtained are an indirect indicator of lattice distortion, micro ε, and anisotropic growth. Though it cannot determine the surface chemical state and the electron defects, the uniformly steady variations in the lattice parameters (a_(100)_ and c_(002)_) correlating to ‘D, ρ_(dislocation)_, TC_(hkl)_, and morphology parameters establish an indication for defect-sensitive growth at 45 °C. The data obtained from this structural parameter are sufficiently robust to justify structure–property relationships for photocatalytic activity without direct measurement of the surface chemical state. The defect-rich ZnO NPs are expected to exhibit enhanced photocatalytic activity due to improved charge separation.^88^

### 3.3 Energy Dispersive X-ray Spectroscopy (EDX)

EDX spectroscopy reveals distinct variations in elemental concentrations in ZnO NPs with growth temperature. In the EDX spectrum of ZnO NPs, the peaks observed at 1.0, 8.6, and 9.5 keV are for Zn, and at 0.5 keV are for O, which are in accordance with the emission peaks of Zn and O atoms.

The EDX spectra indicate the absence of detectable amounts of elemental impurities within the detection limits. The variations in the value of the Zn:O ratio are explained by the presence of lattice stresses generated by heat and variations in the efficiency of O incorporation during the formation of the NPs. There is a steady decrease in the O weight percentage with increasing synthesis temperature. Figure 4 shows the EDX spectra for the ZnO NPs synthesized at different temperatures. Table 4 below shows the elemental composition of the synthesized ZnO NPs at different temperatures, expressed in both weight and atomic percentages.

**Table 4:** Elemental Composition of ZnO NPs Synthesized at Varying Growth Temperatures as Determined By EDX Spectroscopy

	**weight %**		**atomic %**	
**reaction temperature**	O%	Zn%	O%	Zn%
**25 °C**	27.5	72.5	60.8	39.2
**35 °C**	23.7	76.3	55.9	44.1
**45 °C**	24.1	75.9	56.5	43.5
**55 °C**	23	77	55	45
**65 °C**	10.5	89.5	32.5	67.5

**Figure 4:**
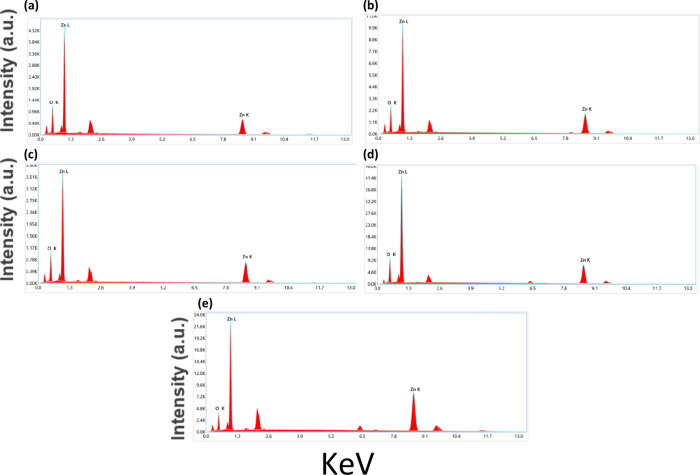
EDX patterns of ZnO NPs with growth temperature at (a) 25 °C, (b) 35 °C, (c) 45 °C, (d) 55 °C, and (e) 65 °C.

Rather than determining the identity of defects such as OV, EDX provides semiquantitative compositional information. OVs and the resulting oxygen deficiency can enhance photocatalytic activity by increasing light absorption, reducing electron–hole recombination rates, and lowering surface energy barriers to charge-transfer kinetics.^89^ OVs and surface states can introduce intermediate energy levels within the E_g_. Defect states enable sub-E_g_ transitions, allowing the absorption of visible light. Synthesis techniques introduce defects; moreover, changes in reaction temperatures promote the formation of O defects, and morphological changes in ZnO, from nanosheets to quasi-spherical NPs, increase the surface area. Additionally, it increases light scattering and trapping in the ZnO NPs. Lattice defects, high surface energy, reduced effective E_g_, and surface states induce electronic perturbations, thereby increasing visible-light photocatalytic activity.^5,62^

Interestingly, the lowest oxygen content is recorded at 65 °C; however, this sample exhibits a low photocatalytic response. The results suggest that excessive nonstoichiometry may introduce deep-level defect states that act as recombination centers, offsetting the benefits of increased O deficiency. This contradictory behavior indicates that the spatial distribution of OVs strongly influences photocatalytic efficiency, particularly their location on the surface and within the subsurface. Subsurface OVs may not effectively contribute to surface redox reactions and instead act as recombination centers for photogenerated charge carriers, thereby limiting photocatalytic activity.^89^

### 3.4 Brunauer–Emmett–Teller (BET) Surface Area Analysis

The variation in reaction temperature significantly influences the BET, specific surface area (S_(BET)_), particle size, and morphology of the synthesized ZnO NPs. The S_(BET)_ (m^2^/g) of the samples was determined from nitrogen (N_2_) adsorption and desorption isotherms. Before analysis, all samples were degassed at 150 °C for 12 h to eliminate residual air and humidity, ensuring an accurate assessment of surface characteristics relevant to photocatalytic performance.

BET analysis was conducted on ZnO NPs synthesized at 25 °C, 35 °C, 45 °C, 55 °C, and 65 °C, which exhibited morphological transitions. The N_2_ adsorption and desorption isotherms exhibit IUPAC Type IV behavior with H3-type hysteresis loops, characteristic of mesoporous materials composed of pores formed by aggregated, nonrigid particles. Figure 5a–e presents the adsorption and desorption isotherms (volume adsorbed vs relative pressure) and corresponding pore size distribution curves for these samples. The main pore-size distribution is in the mesoporous region, with small pores approaching the lower limit of detection, indicating interparticle voids rather than pores within the NPs themselves. The results obtained from BET analysis for pore size distribution were in the region of 1.59–48.32 nm for the 25 °C samples, 1.6–42.84 nm for the 35 °C samples, 1.6–48.82 nm for the 45 °C samples, 1.6–42.57 nm for the 55 °C samples, and 1.6–43.1 nm for the 65 °C samples. The small pores indicate NPs packed tightly together with voids within the interparticle regions of early stage aggregates, which can indirectly indicate temperature-related kinetic rates of NP formation.^90^

**Figure 5:**
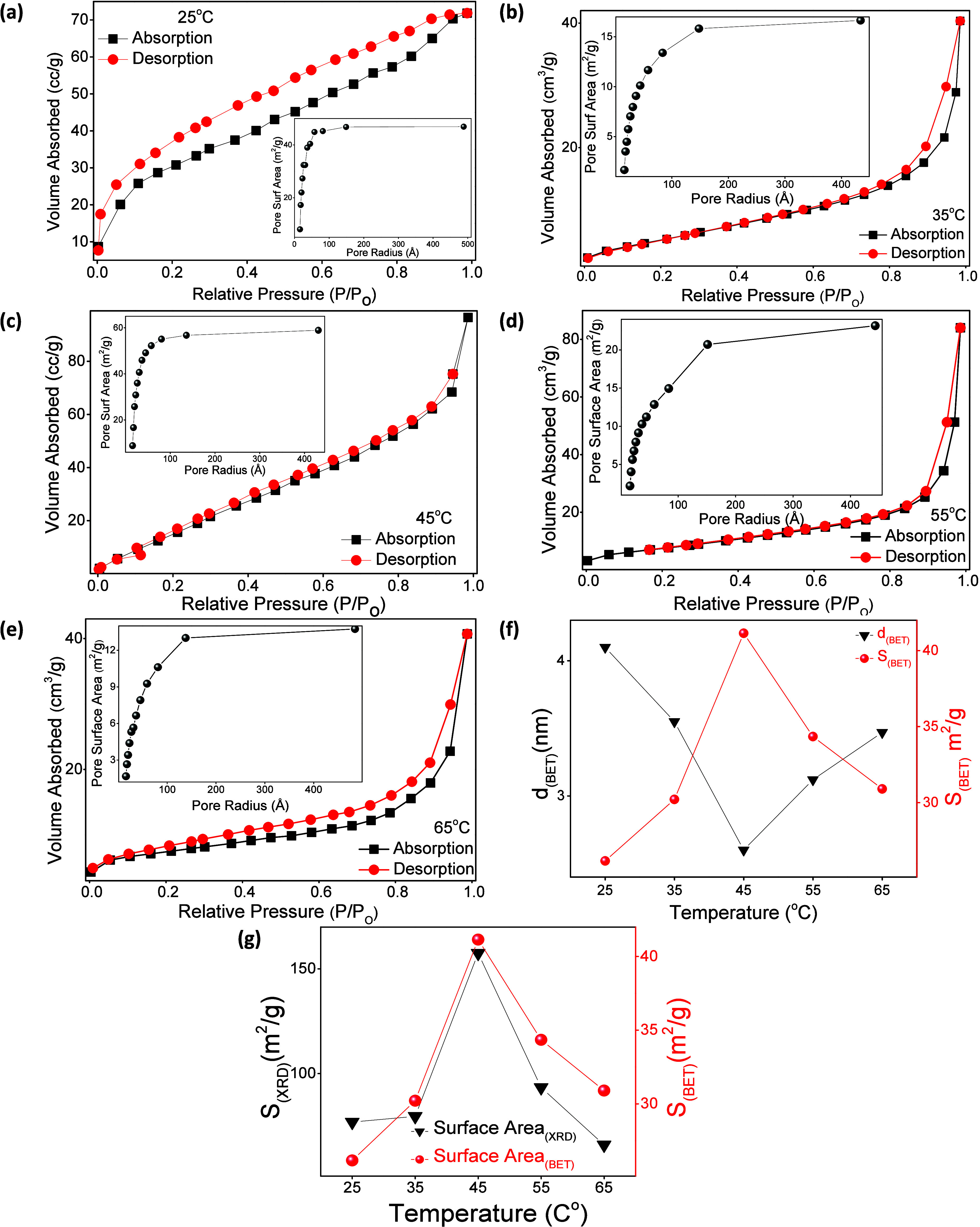
BET adsorption–desorption isotherms, (insets) corresponding pore size distribution plots of ZnO NPs synthesized at (a) 25 °C, (b) 35 °C, (c) 45 °C, (d) 55 °C, (e) 65 °C, (f) comparison of BET-specific surface area (S_(BET)_) and d_(BET)_, and (g) correlation between S_(XRD)_ and S_(BET)_ of ZnO NPs.

The BET-equivalent diameter (d_(BET)_) (nm) was calculated by eq 16,

$$d_{\left(BET\right)}=\frac{6000}{{\rho_{\left(sample\right)}}^{*}S_{\left(BET\right)}}$$ 16

The calculation of d_(BET)_ assumes that the density of ZnO (ρ_(sample)_) (5.6 g/cm^3^),^82^ which may introduce minor deviations for porous nanostructures; however, the values remain suitable for comparative analysis across samples.

Figure 5f presents a comparative analysis of the BET equivalent particle d_(BET)_ and S_(BET)_ for ZnO NPs synthesized at various temperatures. The calculated d_(BET)_ values were 4.1, 3.55, 2.6, 3.12, and 3.47 nm for samples synthesized at 25 °C, 35 °C, 45 °C, 55 °C, and 65 °C, respectively. The d_(BET)_ represents an effective diameter calculated from S_(BET)_ assuming nonporous particles and therefore reflects surface area weighted dimensions rather than true particle size. Correspondingly, the S_(BET)_ values were determined to be 26.158 m^2^/g, 30.211 m^2^/g, 41.141 m^2^/g, 34.343 m^2^/g, and 30.898 m^2^/g. Although the S_(BET)_ values are lower than commercial TiO_2_ P-25 (54 m^2^/g).^91^ The photocatalytic efficiency is governed by a combined effect of surface area, defect density, morphology, and charge-carrier dynamics rather than surface area alone.

Figure 5g expresses the correlation between S_(XRD)_ and S_(BET)_ of ZnO NPs. The divergence between S_(XRD)_ and S_(BET)_ arises from NPs agglomeration and interparticle necking, where multiple crystallites contribute to a single surface accessible entity during N_2_ adsorption. These BET findings are consistent with SEM observations, which reveal pronounced morphological transformations of NPs across the different synthesis temperatures. The significant maximum S_(BET)_ at 45 °C correlates with reduced d_(BET)_ and defect rich morphology, providing a structurally optimized balance between surface accessibility and crystallinity relevant to photocatalytic activity.^82,90^

### 3.5 Ultraviolet–Visible Spectroscopy

UV–visible spectroscopy was used to investigate the temperature-dependent changes in the optical absorption edge and electronic transition behavior of ZnO NPs. Excitonic effects are intrinsic to ZnO, especially near the band edge (NBE). However, the present study focuses on the modulation of the absorption edge rather than on direct exciton dynamics.

Figure 6a shows the absorption spectra. These clearly demonstrate a redshift and widening of the absorption edge relative to bulk ZnO (around 375 nm), indicating increased sub-E_g_ absorption. The redshift is due to changes in the ZnO absorption edge from lattice disorder and defect-related states. The smooth onset of absorption and reddening is linked to the extended absorption edge, a characteristic feature of Urbach-type disorder in semiconductors. Tail absorption arises from structural ε values, disorder, and defect levels, thereby increasing the material’s ability to absorb visible light. The NBE band transition in ZnO is due to the promotion of valence electrons to the direct conduction band (CB). ZnO is a wide E_g_ material with the E_g_ ≈ 3.3 eV.

**Figure 6:**
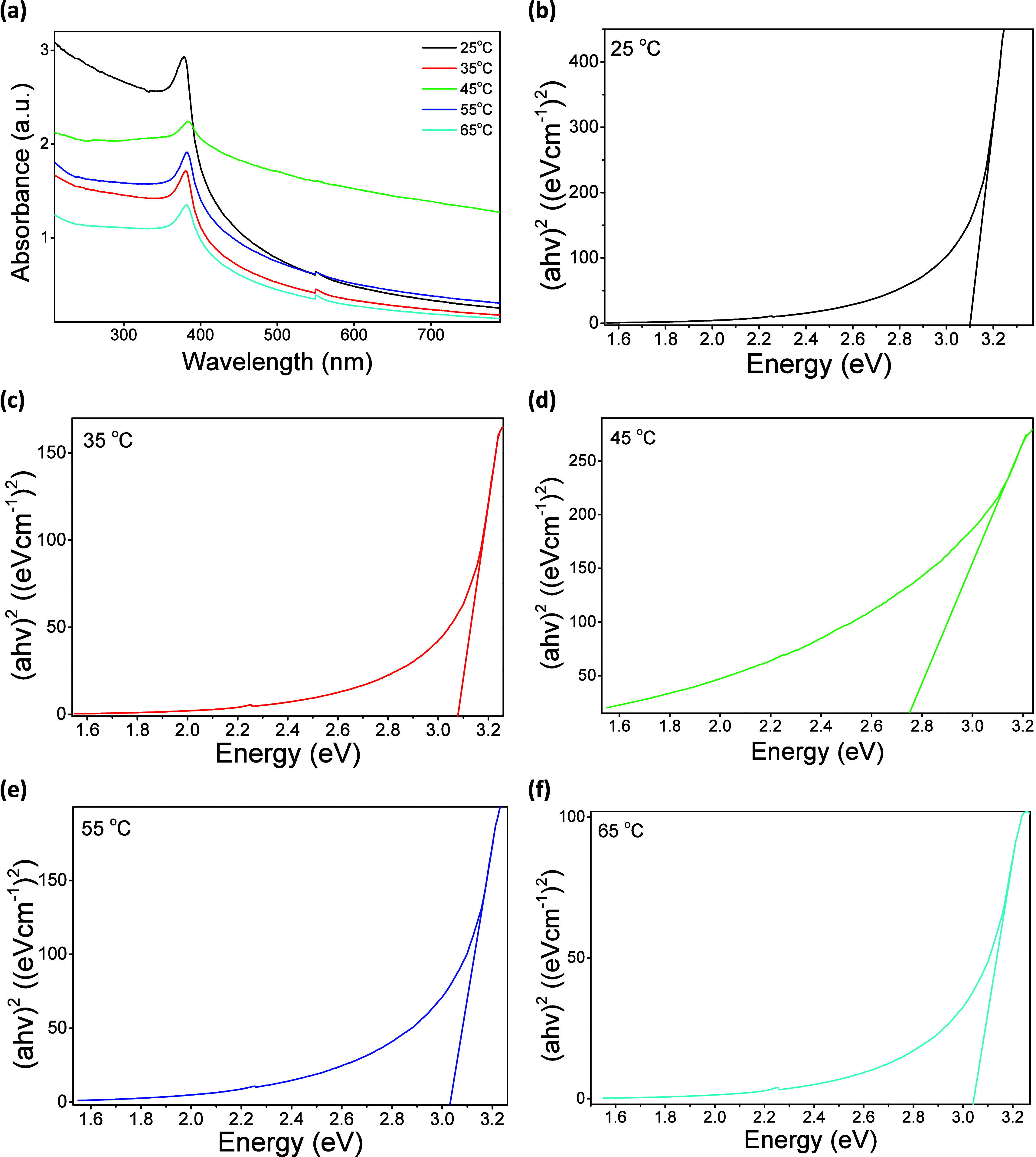
Optical characterization of ZnO NPs synthesized at various temperatures: (a) UV–Vis absorption spectra, Tauc plots for direct bandgap (E_g_) estimation (b) 25°C, (c) 35 °C, (d) 45 °C, (e) 55 °C, and (f) 65 °C.

The ZnO absorption shift with particle size, crystallinity, defect states, impurity concentration, and surface chemistry shifts in lambda max (λ_max_).^64,72^ This spectral shift reflects subtle variations in the E_g_ energies of ZnO NPs synthesized at different growth temperatures. Figure 6b–f exhibits the estimated E_g_ using Tauc’s method, described by eq 17:

$${\left(\mathrm{\alpha h\nu}\right)}^{\gamma }=A\left(h\nu -E_{g}\right)$$ 17

Where α is the absorption coefficient, h is Planck’s constant (6.6261 × 10^–34^ J·s), ν is the photon frequency, γ denotes the type of electronic transition, A is a constant, and E_g_ is the band gap. The value of γ varies by material: γ = 2 for direct allowed, 1/2 for indirect allowed, ^2^/_3_ for direct forbidden, and ^1^/_3_ for indirect forbidden transitions.^92^ For ZnO, a direct E_g_ semiconductor, (αhν)^2^ versus photon energy plots (Figure 6b–f) were used to find the optical E_g_.

E_g_ values for ZnO NPs made at 25 °C, 35 °C, 45 °C, 55 °C, and 65 °C were 3.1, 3.08, 2.75, 3.03, and 3.04 eV. These are lower than the bulk ZnO value (∼3.3 eV), indicating strong size- and surface-dependent effects on the electronic properties. Notably, the 45 °C sample showed the lowest E_g_ (2.75 eV) due to the presence of dense defect states.

The obtained E_g_ values reflect apparent optical E_g_ values affected by sub- E_g_ absorption, rather than the genuinely contracted E_g_ of ZnO NPs at 45 °C. Crystal defects induce the NBE bands, causing a virtual shift of the absorption edge to lower energies, thereby decreasing the apparent E_g_ in a Tauc’s analysis. The most significant decrease in E_g_ occurs in ZnO NPs produced at 45 °C, accompanied by reductions in ‘D and lattice ε, along with a high defect concentration, as revealed by the structural analysis. This correlation supports a relationship between structure and optical properties, highlighting how temperature effects on growth dynamics govern both lattice disorder and optical absorption behavior.

Among the several known variables affecting the optical properties of ZnO, variations of absorption edge values recorded herein are more likely due to lattice disorder, defect states, and electronic perturbations caused by ε. All samples use a similar synthesis method and precursor. As a result, these effects are more dominant than compositional effects. Natural defects enhance sub-E_g_ light absorption. This makes it easier to create exciton pairs under visible-light illumination. E_g_ values increase with reduced grain size due to quantum confinement effects.^93,94^ However, this is more likely due to lattice disorder, lattice ε, natural lattice defects, and changes in carrier concentration. These factors affect the absorption edge.^95^

It should be noted that UV–visible spectroscopy cannot determine defect concentration and defect types. Eg narrowing and absorption tailing are expected when localized states are associated with defects. This is especially true given the structural disorder and small crystallites evident in the XRD studies.

### 3.6 Photoluminescence (PL) Spectroscopy

The PL spectra of ZnO NPs obtained at different temperatures have similar bands but differ in their optical properties, which can be attributed to defect-related emissions (Figure 7). The sharp emission peak at 390 nm corresponds to the NBE. There is also a wide band in the visible region, which is attributed to the presence of intrinsic defects in the ZnO crystal structure. ZnO is known to exhibit strong UV emission due to charge recombination; therefore, incorporating defects into the structure changes the lattice potential, allowing electron transitions into the visible region.^96^

**Figure 7:**
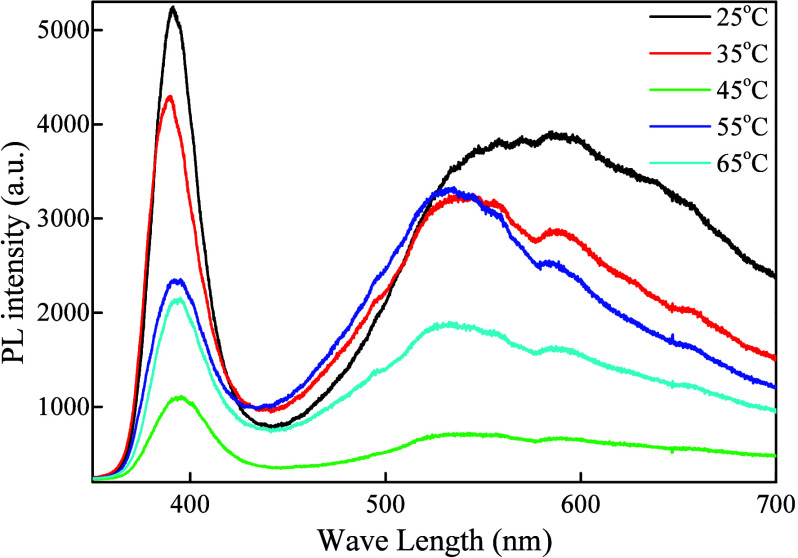
PL spectra of ZnO NPs synthesized at different temperatures.

The relative intensities of UV and visible emissions provide insight into defect densities and morphological changes in ZnO NPs. A blue shift in the NBE emission for samples synthesized at 25 and 35 °C is more logically explained by reduced lattice disorder, lower defect density, ε-related effects, and variations in particle morphology. The UV emission intensity is associated with direct band-to-band exciton recombination, but this process becomes less effective as the synthesis temperature increases. The UV emission is suppressed in the sample synthesized at 45 °C, indicating reduced radiative recombination; however, the observed trend reflects more efficient nonradiative pathways via defect-related trap states, which may be responsible for charge separation. Combining these with efficient surface reaction kinetics is imperative for high photocatalytic activity.

The Stokes shift of approximately 122 meV is observed; the difference between the absorption edge and the PL peak increases with increasing synthesis temperature up to 45 °C. Represents the overall role of exciton coupling, lattice relaxation, and the presence of localized states, in addition to nonradiative relaxation. Variation in PL peak spectral properties and synthesis temperature can indicate a nonlinear defect-formation process that peaks at an intermediate synthesis temperature. The visible PL emission is typically considered to result from deep-level states, such as oxygen-related defects or zinc interstitials in ZnO, which act as midgap states located in the range from the CB to the valence band (VB).^97^

The synthesis temperature generates defect-related localized energy states, leading to optical absorption in the visible region and the formation of intermediate energy levels that may be involved in charge trapping and delayed recombination rate.^58,96^ These defects increase optical absorption in the visible region and help suppress charge recombination, thereby promoting the formation of reactive sites in the photocatalytic redox reaction. Although PL results indicate a change in recombination dynamics due to defect-related energy states, their role in determining photocatalytic properties should be carefully interpreted in relation to optical absorption characteristics and the structure. They are primarily used to control the electronic properties of ZnO NPs to enhance photocatalytic activity under solar radiation. Although PL spectroscopy is helpful for determining pathways for recombination and defect-related emission, a careful analysis is needed to determine defect concentrations and carrier lifetimes.

The excitation conditions for the prepared sample were identical across all PL spectra. This ensures that observed changes in PL intensity reflect intrinsic material properties. PL intensity itself does not provide a quantitative measure of structural defect density. Instead, it reflects the balance between radiative and nonradiative recombination pathways, which are differently affected by structural disorder, surface states, and defect-related trap levels.

### 3.7 Zeta (ζ) Potential

The interfacial behavior of the prepared ZnO NPs upon aqueous suspension was assessed by means of zeta (ζ) potential measurements. ζ-potential gives information about the surface charge of NPs at the solid–liquid interface and determines the electrostatic repulsion or attraction between particles in colloidal dispersions. Upon the application of an electric field, charged particles experience electrophoretic motion, where the direction and velocity of migration are determined by the sign and magnitude of the surface charge as well as the viscosity and dielectric properties of the medium.^98^

ζ-potential magnitude is beyond ± 30 mV normally regarded as indicators of electrostatically stable colloids. This condition is system-dependent and influenced by particle size, ionic strength, and surface chemistry. On the other hand, suspensions with ζ-potential magnitudes below the threshold are susceptible to incipient instability and aggregation, especially for suspensions containing particles of smaller sizes, where surface energy and van der Waals forces dominate. As the particles in nanoscale ZnO become smaller in size, the ζ-potential does not vary monotonically due to the influence of surface •OH group concentration, surface defects, and adsorbed ions. This difference in surface chemistry leads to lower ζ-potential magnitudes, thereby increasing the tendency for particle agglomeration due to decreased repulsive forces.^98^ On the other hand, a higher tendency for agglomeration at higher synthesis temperatures can be inferred from the ζ-potential measurements.

The pH of the solvent medium is a crucial factor in determining the ζ-potential, as it affects the ZnO surface chemistry and ionization equilibrium at the ZnO NPs interface. ZnO NPs tend to have •OH functional groups on their surfaces, and this results in the formation of a neutral layer of Zn–OH at the interface in a neutral solution. However, in an acidic environment, since hydrogen ions (H^+^) are more abundant, the formation of Zn–OH_2_^+^ functional groups are promoted, and this results in a net positive charge being present on the surface of ZnO NPs. On the other hand, in a basic environment, an excess of •OH deprotonates them, enabling the formation of Zn–O^–^ functional groups and generating a negatively charged surface. These surface transformations underscore the pH-dependent nature of ZnO NP stability and surface charge dynamics in colloidal systems.^98^

All ζ-potential analyses were carried out under identical aqueous conditions to ensure that differences in measured ζ-potential were not due to pH variations but to differences in inherent surface properties. The ZnO NPs prepared had a negative ζ potential, indicating that the particles were produced under basic conditions. The negatively charged surface potential of the ZnO NPs can be attributed to the deprotonation of surface •OH functionalities to yield Zn–O^–^ bonds in basic conditions. It is also important to note that, for ZnO NPs, the surface potential decreased with increasing synthesis temperature, accompanied by decreases in particle-liquid interface stability. The increased instability of the particle-liquid interface reduces the photocatalytic efficiency of semiconductor materials by decreasing access to active surface sites.^72^ The decreased ζ-potential values with increasing synthesis temperature of the NPs suggest decreased electrostatic stabilization of the particles, coupled with increased particle agglomeration. Decreased interfacial stability at the particle-liquid interface reduces the semiconductor’s photocatalytic activity by limiting access to active surface sites.

Among the samples tested, the ζ-potential of the ZnO NPs synthesized at 45 °C was – 24.93 mV. Even if it was slightly below the ± 30 mV threshold required to create a stable colloidal dispersion, it was enough to suspend the particles. Even if the ζ-potential of – 24.93 mV was slightly below the ± 30 mV threshold, which was enough to stabilize a colloidal dispersion, it still indicates a moderate degree of stabilization due to electrostatic forces, apart from being morphologically favorable with a large surface area. This sample had the largest active surface area, which was an added advantage.

However, the samples synthesized at 25 and 35 °C exhibit higher ζ-potential values (Table 5), indicating greater colloidal stability. However, the lower photocatalytic activity could be better accounted for by limited accessible surface area, fewer reactive sites available for interaction with OP reactive species, and morphology-dependent active-site availability rather than interfacial charge effects alone. The observation underlined that, while electrostatic stability is vital, photocatalytic performance in ZnO NPs is more crucially controlled by the availability of accessible active surface sites and structural properties. These observations indicate that the ζ-potential alone does not control photocatalytic activity; instead, photocatalytic performance results from contributions from surface charge, accessible surface area, defect density, and charge-carrier dynamics.

**Table 5:** Variation in Zeta (ζ) Potential of ZnO NPs as a Function of Synthesis Temperature

**s no.**	**name**	**temperature (°C)**	**zeta (ζ) potential (mV)**
1	ZnO2	25	–27.43
2	ZnO3	35	–27.43
3	ZnO4	45	–24.93
4	ZnO5	55	–18.44
5	ZnO6	65	–17.31

### 3.8 Photocatalytic Degradation of Rhodamine B (Rh–B)

Rh–B was employed as a probe organic dye to assess the photocatalytic performance of the synthesized ZnO NPs. The photocatalytic degradation was monitored under visible-light irradiation for 200 min. For each ZnO sample, three independent photocatalytic degradation experiments were performed. The mean values of these replicates have been used to create the degradation plots reported in this study, which ensures that the results are statistically reliable and can be reproduced.

To rigorously analyze the degradation behavior, the experimental data were interpreted using two complementary kinetic approaches. First, the degradation profiles were fitted using a nonlinear exponential decay model by eq 18 ^99^ in Figure 8a:

$$A\left(t\right)=Xe^{-k_{ap}t}+E$$ 18

Where A­(t) is the absorbance at time t, X is the fraction of Rh–B undergoing photocatalytic degradation, *k*_ap_ is the apparent degradation rate constant, and E is the residual absorbance. This is due to the presence of stable intermediates, surface-bound species, and light-scattering effects related to the photocatalyst. This nonlinear fitting method prevents distortions caused by the logarithmic linearization of the model and accounts for incomplete mineralization, an intrinsic characteristic of heterogeneous photocatalytic reactions. The inclusion of a nonzero residual term indicates that, in realistic operating conditions, the complete disappearance of optical absorbance is not possible due to surface interactions and intermediate formation.^99^ The inclusion of a nonzero residual of Rh–B indicates that, in realistic operating conditions, the complete disappearance of optical absorbance is not possible due to surface interactions and intermediate formation.

**Figure 8:**
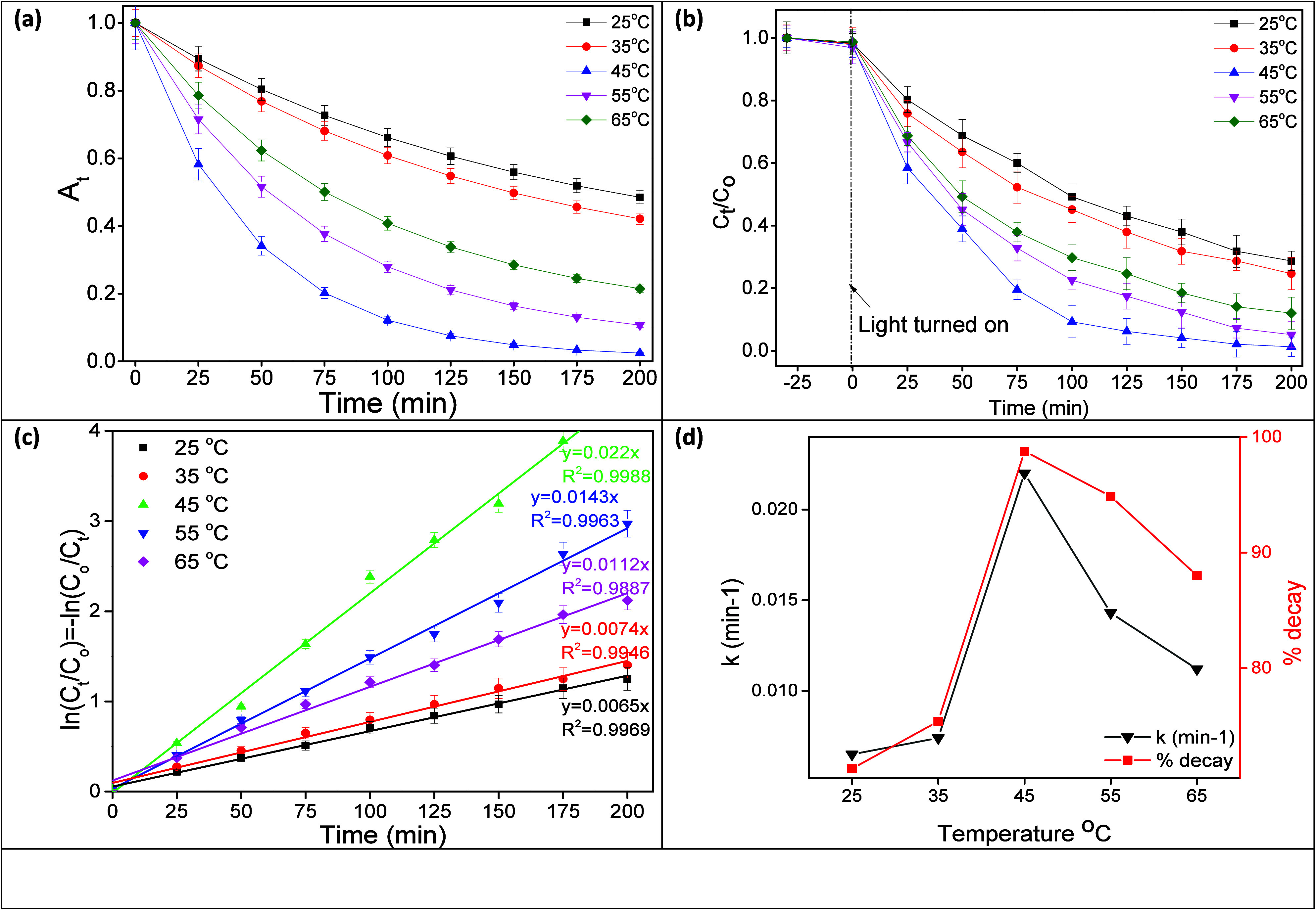
Photocatalytic degradation profiles of Rhodamine B (Rh–B) under visible light irradiation; (a) nonlinear least-squares fitting C_t_ vs time (b) C_t_/C_o_ vs time (c) pseudo-first-order kinetic fits (ln (C_o_/C_t_) vs time); (d) comparison of degradation % decay and rate constants (k, min^–1^) for ZnO NPs synthesized at varying growth temperatures.

The exponential decay profile is consistent with the dominant role of surface photoinduced processes in Rh–B degradation rather than mass transport-limited kinetics. However, *k*_ap_ is considered an apparent kinetic constant, since the overall rate of degradation is a convolution of photon absorption, charge-carrier generation, defect-mediated charge separation, interfacial charge transfer, and surface redox reactions. An absorbance plateau indicates deviations from ideal first-order kinetics at longer irradiation times, consistent with partial surface coverage by reaction intermediates and saturation of active sites, rather than catalyst deactivation, as is typical of immobilized photocatalytic systems.^48,51^

For comparative benchmarking with literature reports, the degradation results were further analyzed by a pseudo-first-order kinetic model, plotted as C0/C_t_ and ln­(C0/C_t_) against time (Figure 8b,c). Linear regression of the plots provided apparent rate constants of 0.0065, 0.0074, 0.022, 0.0143, and 0.0112 min^–1^ for ZnO samples prepared at 25 °C, 35 °C, 45 °C, 55 °C, and 65 °C, respectively. The degradation efficiencies were 71.3%, 75.4%, 98.76%, 94.87%, and 88%, and are compared with the rate constant in Figure 8d, which further verifies that the ZnO prepared at 45 °C has the highest photocatalytic activity. These linearized kinetics serve as a convenient comparative metric rather than a strict mechanistic descriptor, and their consistency with the nonlinear fitting validates the observed activity trends.

The significant photocatalytic performance of ZnO NPs prepared at 45 °C is attributed to synergistic effects of defect engineering, morphology transformation, and optimized crystallinity.^72^ The optimal concentration of OVs and defect-related states, which can enhance visible light absorption and facilitate charge separation, was achieved through controlled-temperature synthesis of ZnO NPs.^64^ At the same time, the morphological transformation from nanosheet-like to quasi-spherical shapes can increase the accessible surface area and reactive facets, thereby promoting adsorption and ROS production.^70^ Notably, the crystallinity is still high enough to prevent excessive charge recombination and maintain favorable surface defects, as previously reported in structural and optical characterization studies.^67,72,97^ Table 6 shows that the synthesized ZnO NPs possess superior photocatalytic activity compared to most reported wide E_g_ photocatalysts under visible light irradiation.

**Table 6:** Comparative Photocatalytic Performance of Wide Bandgap (E_g_) Semiconductor NPs for Rhodamine B (Rh–B) Degradation

	**bandgap (**E_g_ **)**	**light source**	**catalyst dose**	**pollutant loading**	**irradiation time**	**degradation efficiency**	**rate constant**	
**photocatalyst**	**(eV)**	**(nm)**	**(g L** ^**–1**^ **)**	**(mg L** ^**–1**^ **)**	**(min)**	**(%)**	**(k, min** ^**–1**^ **)**	**ref**
**ZnO**	3.12	UV	1	50	180	96%		^67^
**CeO**_**2**_ **/ZnO**	3.08	visible	1	5	240	60%	0.024	^100^
**ZnO@SiO**_**2**_		UV	15	15	180	82.5%		^101^
**TiO**_**2**_		visible	5	5	240	98%		^102^
**TiO**_**2**_ **/RGO/PMMA**	2.96	UV	1	3	160	94%		^103^
**chitosan/SnO**_**2**_		visible	5	5	70	95%	0.103	^104^
**CuO-ZnO**	1.40	UV	1	1	105	96%	0.048	^105^
**ZnO NPs (this work)**	2.75 eV	visible	0.4	5	200	98.76%	0.022	

In summary, the joint nonlinear and pseudo-first-order kinetic analysis offers a physically interpretable and statistically valid model for Rh–B degradation by visible-light irradiation. The findings clearly show that synthesis temperature is the major factor influencing photocatalytic activity through defect structure, accessibility, and charge carrier dynamics. The enhanced activity in the absence of heterojunctions, dopants, and cocatalysts emphasizes the inherent merit of defect-engineered ZnO nanostructures for visible-light-driven photocatalysis.

### 3.9 Photocatalytic Mechanism of Rhodamine B (Rh–B) Decay

The synthesis temperature of ZnO NPs induces lattice distortion, micro-ε, peak broadening, and **ρ**_**(dislocation)**_ which are widely correlated with the presence of intrinsic point defects, particularly oxygen-related defects, that influence the electronic structure and charge transport. Therefore, defect-related interpretations in this study are presented as structure property correlations supported by optical and photocatalytic behavior rather than direct defect quantification.^24,97^

The increased photocatalytic efficiency of the ZnO NPs synthesized at 45 °C is related to the specific crystallographic features, as evidenced by XRD analysis. At this temperature, ZnO exhibits optimal lattice ε and **ρ**_**(dislocation)**_, indicating a defect-rich yet structurally coherent framework that facilitates efficient charge-carrier separation. Additionally, the moderate defect concentration, primarily consisting of oxygen related defects, enhances visible-light absorption, facilitating effective electron transfer to surface-adsorbed O_2_ and increasing the formation rate of ROS, superoxide (•O_2_^–^) radicals. Specific oxygen defect species (VO, Zni) are proposed based on well-established correlations reported for ZnO synthesized under similar conditions;^24,38,83^ however, they are discussed here as probable contributors rather than directly confirmed entities.

At lower synthesis temperatures of 25 °C–35 °C, reduced atomic mobility limits crystallite growth and lattice relaxation. which results in smaller crystallites and higher micro-ε rather than true amorphization. The broadening of the XRD diffraction peaks observed at these temperatures is therefore attributed to crystallite-size effects and localized lattice disorder. It can introduce recombination defect sites while reducing the reactive sites without forming fully amorphous ZnO phases.^86^

On the contrary, higher-temperature syntheses (55–65 °C) promote atomic rearrangements and grain coarsening, leading to decreases in lattice ε and TC_(hkl)_ values, which, in turn, reduce the specific surface area and the density of active sites. Such structural consolidation suppresses surface reactivity and limits the photocatalytic interface reactions. The so-obtained ZnO, therefore, synthesized at 45 °C, attains a critical balance between crystallinity, defect density, and morphology for superior photocatalytic efficiency by balancing charge mobility and surface activity. The photocatalytic degradation mechanism of Rh–B by ZnO NPs under visible light irradiation is depicted in Figure 9.

**Figure 9:**
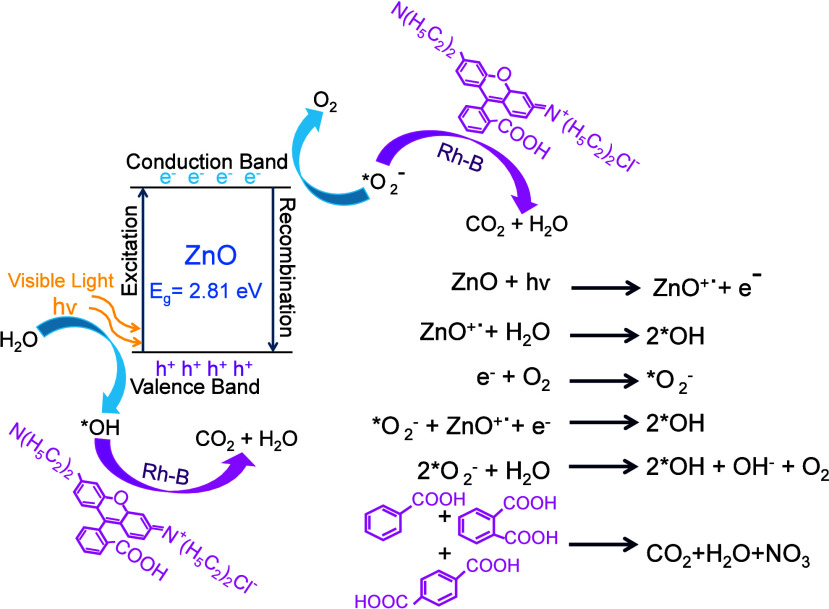
Proposed mechanism for Rhodamine B (Rh–B) degradation under visible light using ZnO NPs, depicting charge carrier dynamics and reactive species pathways.

Although a direct quantitative correlation between individual XRD parameters and photocatalytic rate constants is not established, consistent trends across structural, optical, and kinetic data sets support the proposed structure–property relationship.^24,97^

Under visible-light irradiation, ROS production in ZnO is found to be driven primarily by electrons transfer via surface defect states rather than by band-to-band excitation. The •O_2_^–^ radicals, produced simultaneously with defect-trapped electrons, is the major oxidative species, while the possible mechanisms related to the production of •OH might be surface-mediated defect states. Therefore, ROS production in this case is driven by defect-mediated charge separation, not by wide-E_g_ excitation. These ROS are primarily responsible for the oxidative breakdown of Rh–B into CO_2_, H_2_O, and other intermediate products.^64–66,70,72^ In the absence of scavenger experiments, ROS assignments are inferred from band energetics, defect-mediated electron transfer pathways, and prior ZnO literature on Azo dyes photocatalytic under visible-light excitation claimed were taken in account,^24,52,64–66,70,72^ rather than the repeating the direct experimental proof.

The pH of the photocatalytic studies was maintained at 9.26 at the start of the reaction and decreased to 8.66 upon completion. The pH decrease is interpreted only as a secondary indicator of intermediate formation and reaction progression, not as evidence of complete mineralization. Moreover, the reaction temperature was maintained at 25 °C to eliminate thermal effects and ensure that the observed kinetics arise photocatalytic decay only. The decrease in pH during photocatalysis reflects the formation of acidic intermediate species and the progressive oxidative degradation of Rh–B but does not provide direct proof of mineralization. While pH variation indicates ongoing photocatalytic reactions thus, pH trends are used in the present study as secondary indicators of the progression of degradation.

In addition to the intrinsic photocatalytic activity, structural defects particularly abundant at intermediate synthesis temperatures, introduce shallow trap states within the E_g_. These defect states extend light absorption into the visible region and enhance charge carrier separation by suppressing rapid recombination, thereby improving quantum efficiency. The enhanced light harvesting in the visible spectrum is thus partially attributed to sub-E_g_ transitions facilitated by these surface and lattice defects.^64,66,72^

Moreover, the degradation of Rh–B may proceed via a dye-sensitization pathway process under visible-light illumination. In this mechanism, Rh–B molecules first absorb visible light and transition to an excited state (Rh–B*), from which electrons can be injected into the CB of ZnO. These injected electrons contribute to the generation of •O_2_^–^ radicals, thereby augmenting the photocatalytic process, especially under longer-wavelength visible light.^72^ The dye-sensitization pathway is discussed as a possible auxiliary contribution; however, the strong correlation between photocatalytic performance and synthesis-temperature-dependent ZnO properties suggests that intrinsic photocatalysis dominates. This effect is a synergistic contribution rather than a dominant effect, since both photocatalysis and structural imperfection remain active across thicker cycles of illumination and are well-correlated with synthesis temperature. The observed improvement at 45 °C thus appears to arise from optimized catalyst properties rather than a dye-sensitization effect.

Kinetic analysis of the degradation process, modeled by pseudo-first-order kinetics, revealed that the sample synthesized at 45 °C exhibited the highest rate constant (k = 0.022 min^–1^), which correlated with its enhanced surface area, optimal crystallinity, and defect-assisted charge transport. These findings underscore the critical interplay between NP morphology, defect chemistry, and charge dynamics in tuning photocatalytic performance.^64^

The pseudo-first-order kinetic model employed in this study serves as a comparative tool for evaluating photocatalytic performance under identical experimental conditions and does not imply a definitive reaction mechanism. The extracted reaction rate constants reflect the combined influence of light absorption by the photocatalyst, surface reactions, defect-assisted charge transport, and adsorption dynamics. Mechanistic insights are therefore derived from correlative analysis with structural and optical characterization rather than from kinetic fitting alone.

### 3.10 Photocatalytic Reactor for Rhodamine B (Rh–B) Decay

Rh–B dye as a probe for OPs to study the photocatalytic performance of the reactor under visible light irradiation for 3 h per cycle. The reactor design addresses two significant challenges typically associated with nanoscale photocatalysts: the leaching of powdered catalysts into water systems and the complexity of recovering NPs after reaction. By immobilizing ZnO NPs on the reactor’s internal surfaces, the system enables a portable, reusable, and antifouling water purification platform.

Figure 10 illustrates that the reactor exhibits good reproducibility in successive cycles of Rh–B degradation under visible-light illumination. Consistent degradation efficiencies across cycles confirm the stability of the immobilized ZnO photocatalyst layer, with negligible catalyst deactivation, confirming its structural and interfacial stability under operational conditions.

**Figure 10:**
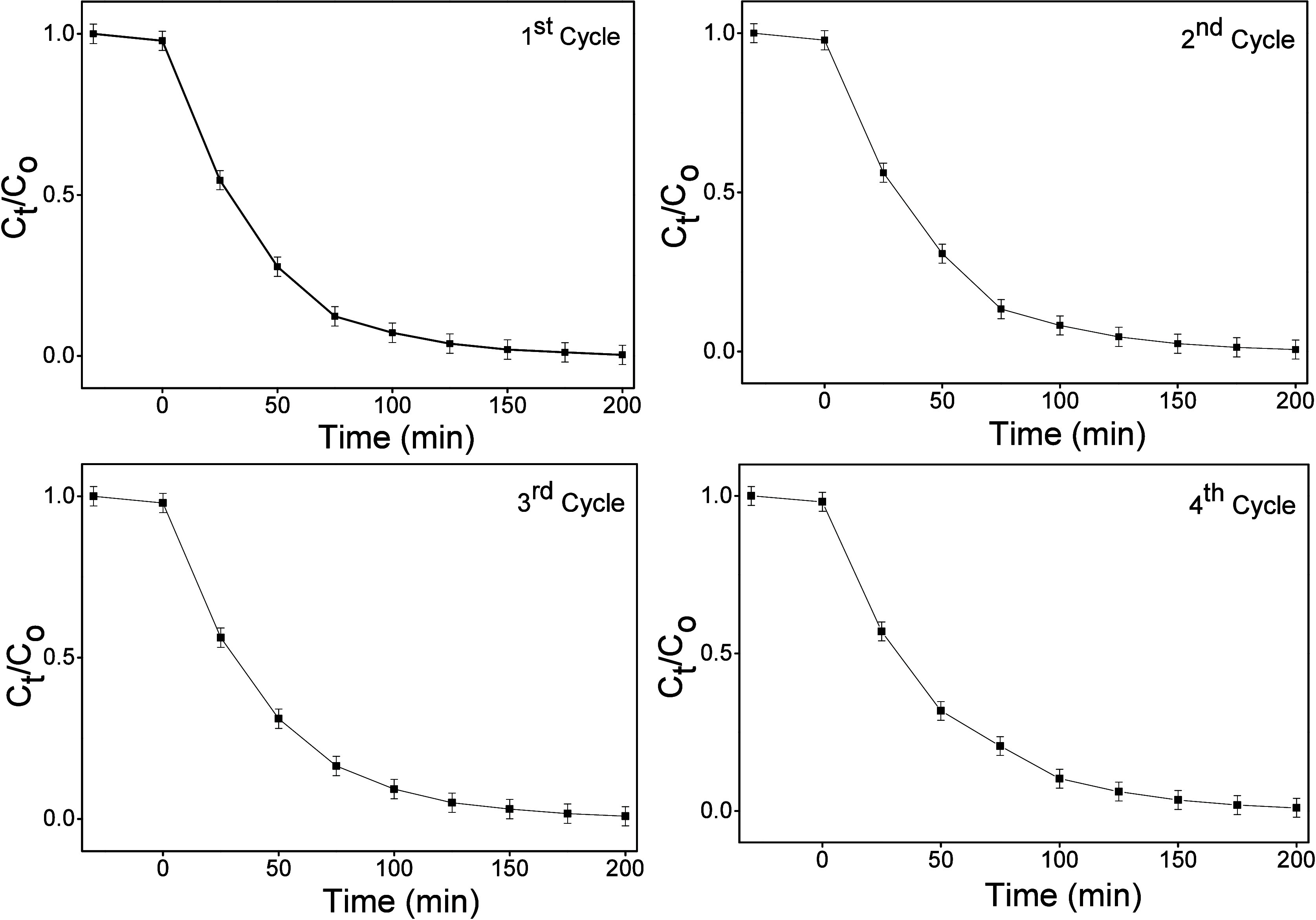
Reusability of ZnO-based photocatalytic reactor for Rhodamine B (Rh–B) degradation under visible light, showing consistent degradation efficiency and kinetic stability across multiple cycles.

Importantly, the four-cycle stability test was designed to assess the intrinsic robustness of the immobilized ZnO photocatalyst rather than to replicate the full operational lifetime of the reactor. In immobilized systems, catalyst deactivation is not dominated by mechanical loss, as in slurry-based configurations, but is instead governed by surface-state evolution, defect stability, and charge-carrier dynamics. The enhanced photocatalytic activity is attributed to efficient light management within the photocatalytic chamber. Total internal reflection significantly enhances visible-light harvesting, while mirror-coated reactor walls generate evanescent-wave excitation, thereby increasing the photon flux at the ZnO interface. These effects collectively enhance electron–hole generation and interfacial charge transfer without increasing external energy input, thereby boosting photocatalytic efficiency.

These trends in stability are inextricably linked to the defect structure of ZnO, which itself depends on the preparation temperature. More specifically, the ZnO prepared at 45 °C has an optimized level of defects and crystallinity that enables efficient charge separation and, crucially, avoids an excessive concentration of deep-level traps that would lead to a rapid nonradiative recombination pathway in other materials. Hence, the stability of the photocatalyst is itself a function of the controlled environment of defects, not merely the number of reuse cycles.

Over prolonged operation, a degradation trend in photocatalytic activity might emerge due to reversible surface-related phenomena, including the adsorption of reaction intermediates, surface hydroxylation of active sites, and defect-state saturation, but not due to lattice degradation. For immobilized ZnO catalysts, these degradation trends can be readily prevented through photocatalytic surface reactivation cycles. This short-term solar irradiation treatment, without any pollutant from clean water, can rejuvenate surface catalytic sites by desorbing surface-adsorbed reaction intermediates and regenerating defect-mediated charge-transfer sites, without affecting crystal structure and coating integrity.

Although testing in continuous flow under long-duration conditions is needed, by considering an immobilized ZnO structure, an optimal synthesis temperature, and reversible surface deactivation, there is compelling evidence that stable, long-term photocatalysts are possible. Remaining challenges for scale-up include maintaining uniformity and controlling the thickness of ZnO coatings within the photocatalytic chamber. Overdeposition of NPs may block active sites and limit the photocatalytic reaction of pollutants. Therefore, optimizing the coating process to ensure balanced exposure and mechanical stability is vital for scaling up the system and maintaining high performance.

This study highlights a promising route for integrating visible-light-responsive nanomaterials into scalable, low-maintenance, and sustainable photocatalytic reactors for advanced water purification applications.

## 4 Conclusion

This work establishes a relationship for ZnO NPs synthesized via a temperature-modulated coprecipitation route, demonstrating how variations in growth temperature govern crystallographic order, defect chemistry, optical behavior, and photocatalytic activity. All samples retained the wurtzite hexagonal phase; however, XRD analysis revealed systematic lattice perturbations with temperature, highlighted by a distinct contraction in the c-lattice parameter at 45 °C. This distortion correlates with a morphological shift from sheet-like structures to quasi-spherical particles, indicating that temperature plays a significant role in the dynamics of nucleation and growth of NPs in defining crystallite architecture.

In this temperature series, a critical transition occurs at 45 °C, where enhanced crystallinity is achieved with moderate lattice ε, an optimized **ρ**_**(dislocation)**_, and a preferential polar facet orientation, as verified by TC_(hkl)_ analysis. These structural features collectively increase the density and accessibility of catalytically active sites. SEM and EDX characterization confirm that a favorable distribution of lattice distortion indeed accompanies morphological changes, a defect state known to enhance charge trapping, interfacial transfer, and ROS generation during photocatalysis.

Optical analysis further confirms the structural optimum at 45 °C. Temperature-dependent UV–vis absorption studies reveal a continuous E_g_ narrowing down to 2.75 eV, consistent with defect-mediated sub-E_g_ transitions and enhanced visible-light harvesting. Correspondingly, a PL quench at this temperature reflects a suppression of electron–hole recombination and increased charge-carrier lifetime consistent with an augmented density of intrinsic defects. BET analysis shows a significant increase in surface area to 41.14 m^2^/g and a hierarchically distributed pore network, enabling superior adsorption-reaction kinetics.

The ZnO NPs fabricated at 45 °C have consistently shown better performance than other samples for Rh–B degradation under visible-light irradiation. Based on the optimized properties, a portable photocatalytic reactor was developed by immobilizing ZnO to prevent NPs leaching, while ensuring photon harvesting via total internal reflection, thereby enabling repeated catalytic cycles with stable activity. While the current approach to deposition is highly feasible, further refinement of coating uniformity and thickness control, along with long-term photostability, is imperative in future work to improve catalytic yield and integration potential for decentralized water treatment systems. In summary, this study provides a robust framework for rationally tailoring ZnO nanostructures through controlled synthesis temperature and demonstrates their practical translation into functional reactor platforms for sustainable water purification.
